# Supplementary material for: Evaluation of CRISPR gene-editing tools in zebrafish
Source: BMC Genomics. 2022 Jan 6;23:12. doi: 10.1186/s12864-021-08238-1 (PMC8734261; doi:10.1186/s12864-021-08238-1)
Supplement: Supplementary file 1 — Additional file 1: Supplementary Figure 1. Editing efficiencies across 50 gRNAs with different GC content. Supplementary Figure 2. Mosaicism predicted using Illumina sequencing of G0 mutant larvae. Supplementary Figure 3. Network analyses of the shared differentially expressed genes in Cas9 enzyme and Cas9 mRNA injected larvae. Supplementary Table 1. Oligonucleotides utilized for all assays performed throughout the project. Supplementary Table 2. On-target efficiency scores for the 50 gRNAs obtained from different in silico prediction tools, empirical cleavage scores in vivo using Sanger sequencing and sequence deconvolution tools (TIDE and ICE), from the in vitro assay CIRCLE-seq (RPMN), and Illumina sequencing using the CrispRVariants software. Supplementary Table 3. Description of the off-targets predicted by CRISPRScan and CIRCLE-seq for the 50 evaluated gRNAs. The percentage of sites intersecting genes from the CRISPRScan tool was obtained from the top 30 predicted off-targets since the tool only provides detailed information from these. Supplementary Table 4. List of the frameshift variants identified in protein-coding genes in samples injected with either Cas9 enzyme or Cas9 mRNA using uninjected batch-siblings as reference. Supplementary Table 5. Illumina mosaicism percentages observed in larvae injected with Cas9 (enzyme or mRNA), catalytically dead Cas9 (dCas9), scrambled gRNA, uninjected batch siblings, and a finclip from their crossing parents. Supplementary Table 6. List of DE genes observed in Cas9-injected samples relative to uninjected batch-siblings and siblings from another batch. Supplementary Table 7. List of gene ontology terms enriched in upregulated genes found in both injection treatments (Cas9 enzyme and Cas9 mRNA). [file 12864_2021_8238_MOESM1_ESM.zip › SupFigures_R1_v2.pdf]

Supplementary Figures for:

Evaluation of CRISPR gene-editing tools in zebrafish

José M. Uribe-Salazar[1,2], Gulhan Kaya[1], Aadithya Sekar[1], KaeChandra Weyenberg[1], Cole Ingamells[1], Megan Y. Dennis[1,2†]

[1]Genome Center, MIND Institute, and Department of Biochemistry & Molecular Medicine, University of California, Davis, CA, USA; [2]Integrative Genetics and Genomics Graduate Group, University of California, Davis, CA, USA

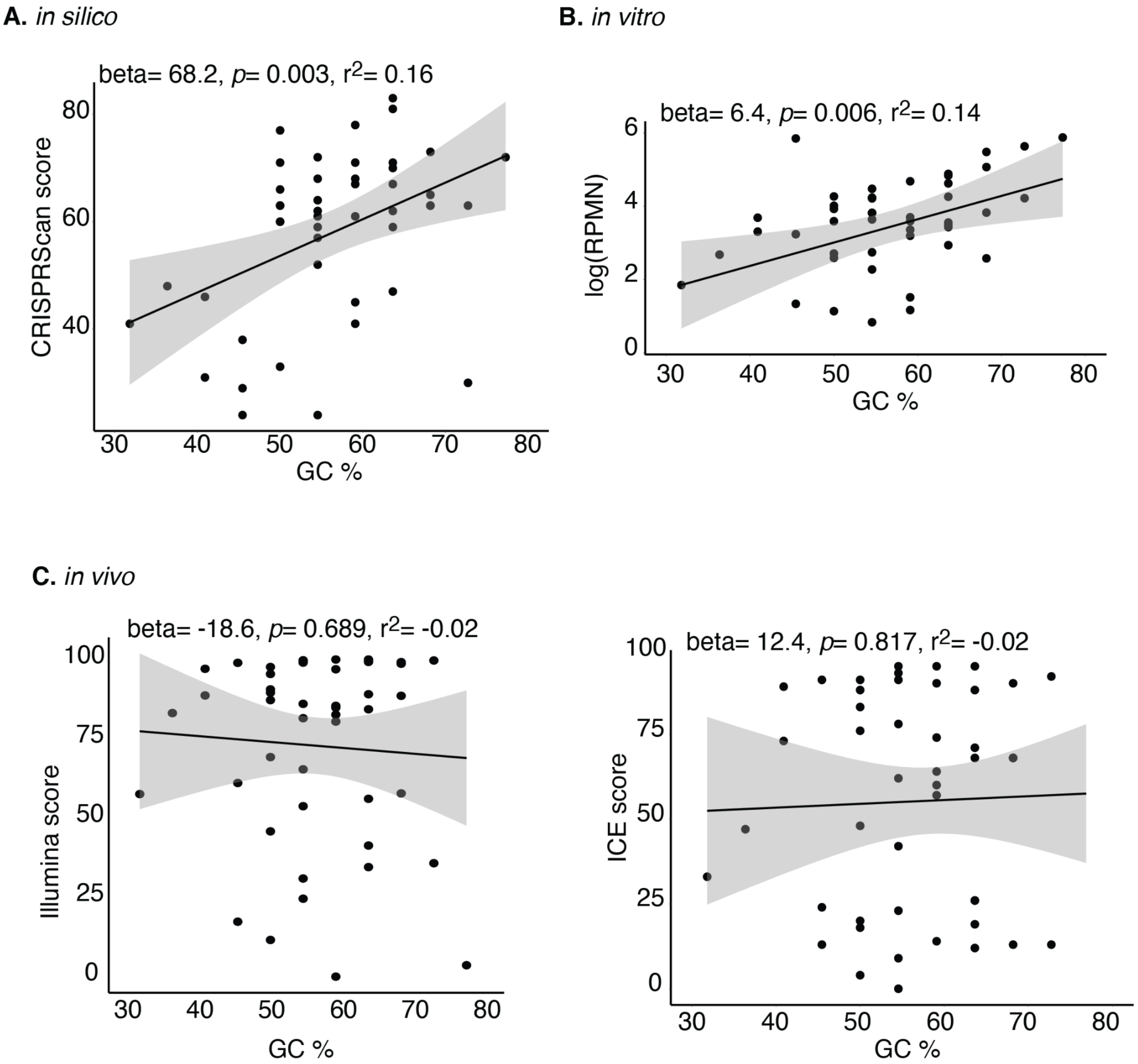

Supplementary Figure 1. Editing efficiencies across 50 gRNAs with different GC content. Editing efficiencies **(A)** predicted *in silico* using the CRISPRScan algorithm, **(B)** predicted *in vitro* using the reads per million normalized (RPMN) from the CIRCLE-seq protocol, and **(C)** estimated *in vivo* with Sanger sequencing and the ICE tool (see Methods for details). Results from linear regressions are shown for each comparison.

Supplementary Figure 2. **Mosaicism predicted using Illumina sequencing of G_0 mutant larvae.** Alleles identified with *CrispRVariants* in larvae injected with Cas9 enzyme (“Cas9enzyme”), Cas9 mRNA (“Cas9RNA”), dCas9 (“dCas9”), scrambled gRNA (“Scrambled”), uninjected batch siblings (“Uninjected”), and a flin-clip from their crossing parents (“Parentals”) across 21 genes. Percentages of reads with identified alleles are shown to the right of each allele sequence, with increasing red shades indicating higher percentages.

actb1:

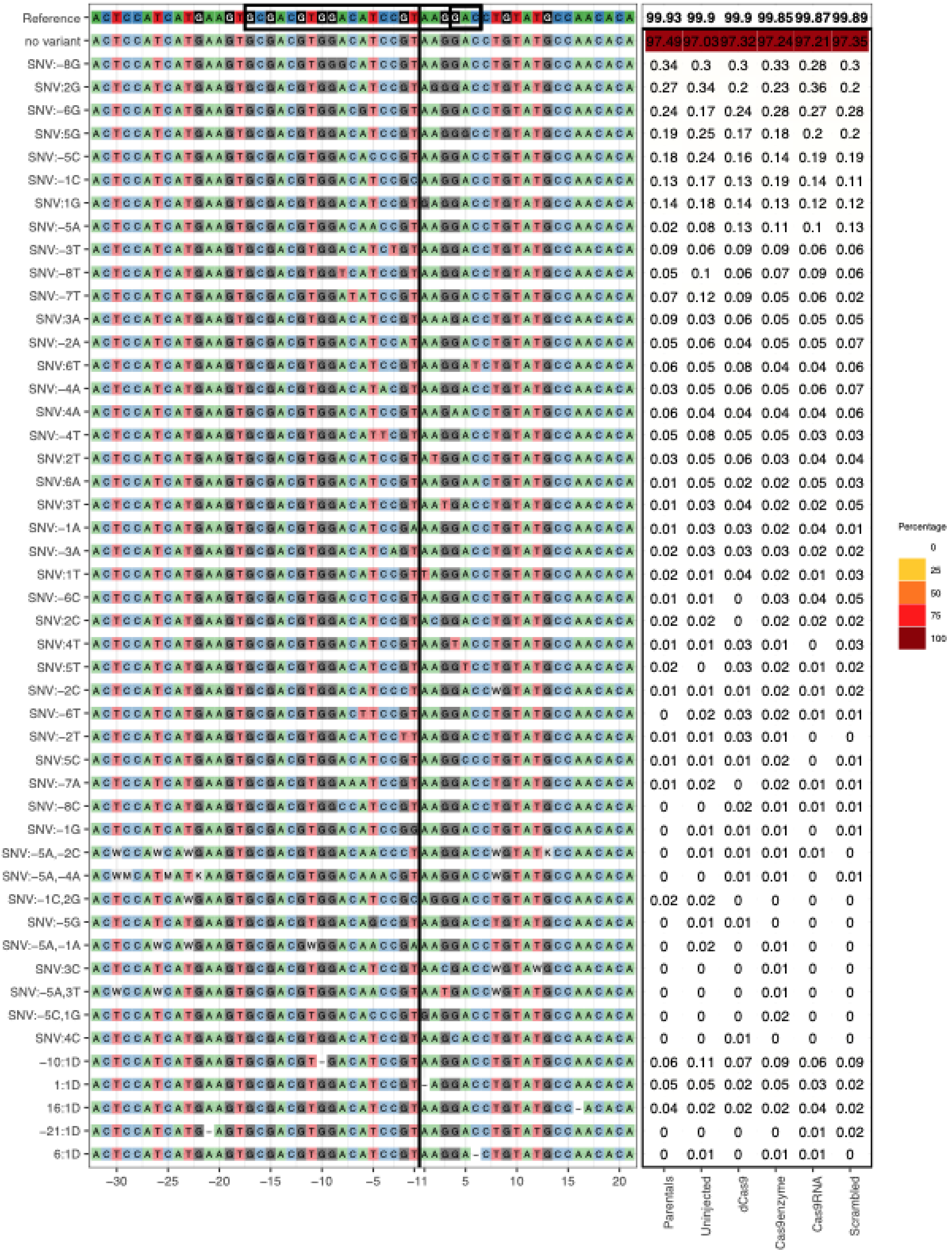

anp32b:

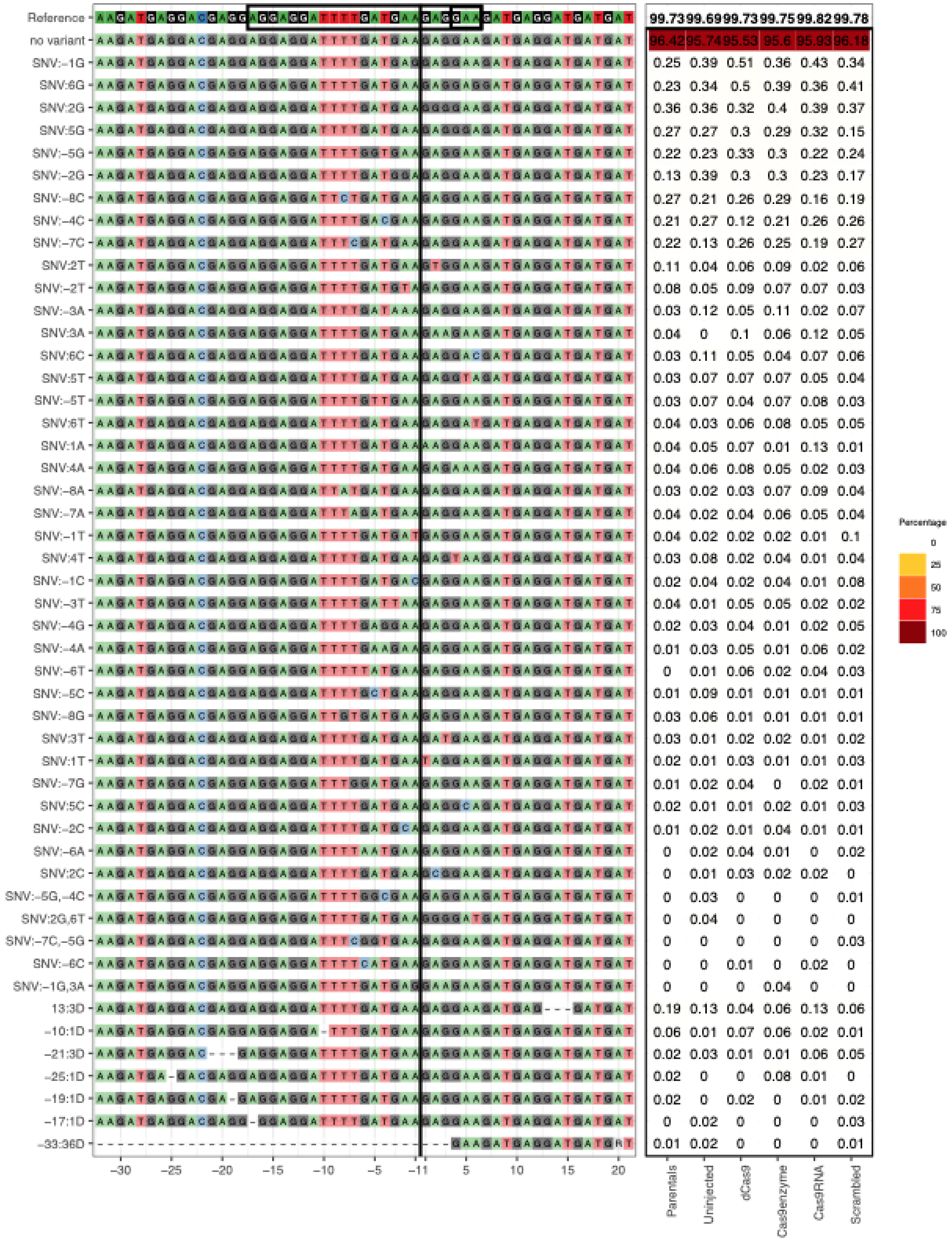

Reference 99.73 99.69 99.73 99.75 99.82 99.78
no variant 96.42 95.74 95.53 95.6 95.93 96.18
SNV:−1G 0.25 0.39 0.51 0.36 0.43 0.34
SNV:6G 0.23 0.34 0.5 0.39 0.36 0.41
SNV:2G 0.36 0.36 0.32 0.4 0.39 0.37
SNV:5G 0.27 0.27 0.3 0.29 0.32 0.15
SNV:−5G 0.22 0.23 0.33 0.3 0.22 0.24
SNV:−2G 0.13 0.39 0.3 0.3 0.23 0.17
SNV:−8C 0.27 0.21 0.26 0.29 0.16 0.19
SNV:−4C 0.21 0.27 0.12 0.21 0.26 0.26
SNV:−7C 0.22 0.13 0.26 0.25 0.19 0.27
SNV:2T 0.11 0.04 0.06 0.09 0.02 0.06
SNV:−2T 0.08 0.05 0.09 0.07 0.07 0.03
SNV:−3A 0.03 0.12 0.05 0.11 0.02 0.07
SNV:3A 0.04 0 0.1 0.06 0.12 0.05
SNV:6C 0.03 0.11 0.05 0.04 0.07 0.06
SNV:5T 0.03 0.07 0.07 0.07 0.05 0.04
SNV:−5T 0.03 0.07 0.04 0.07 0.08 0.03
SNV:6T 0.04 0.03 0.06 0.08 0.05 0.05
SNV:1A 0.04 0.05 0.07 0.01 0.13 0.01
SNV:4A 0.04 0.06 0.08 0.05 0.02 0.03
SNV:−8A 0.03 0.02 0.03 0.07 0.09 0.04
SNV:−7A 0.04 0.02 0.04 0.06 0.05 0.04
SNV:−1T 0.04 0.02 0.02 0.02 0.01 0.1
SNV:4T 0.03 0.08 0.02 0.04 0.01 0.04
SNV:−1C 0.02 0.04 0.02 0.04 0.01 0.08
SNV:−3T 0.04 0.01 0.05 0.05 0.02 0.02
SNV:−4G 0.02 0.03 0.04 0.01 0.02 0.05
SNV:−4A 0.01 0.03 0.05 0.01 0.06 0.02
SNV:−6T 0 0.01 0.06 0.02 0.04 0.03
SNV:−5C 0.01 0.09 0.01 0.01 0.01 0.01
SNV:−8G 0.03 0.06 0.01 0.01 0.01 0.01
SNV:3T 0.03 0.01 0.02 0.02 0.01 0.02
SNV:1T 0.02 0.01 0.03 0.01 0.01 0.03
SNV:−7G 0.01 0.02 0.04 0 0.02 0.01
SNV:5C 0.02 0.01 0.01 0.02 0.01 0.03
SNV:−2C 0.01 0.02 0.01 0.04 0.01 0.01
SNV:−6A 0 0.02 0.04 0.01 0 0.02
SNV:2C 0 0.01 0.03 0.02 0.02 0
SNV:−5G,−4C 0 0.03 0 0 0 0.01
SNV:2G,6T 0 0.04 0 0 0 0
SNV:−7C,−5G 0 0 0 0 0 0.03
SNV:−6C 0 0 0.01 0 0.02 0
SNV:−1G,3A 0 0 0 0.04 0 0
13:3D 0.19 0.13 0.04 0.06 0.13 0.06
−10:1D 0.06 0.01 0.07 0.06 0.02 0.01
−21:3D 0.02 0.03 0.01 0.01 0.06 0.05
−25:1D 0.02 0 0 0.08 0.01 0
−19:1D 0.02 0 0.02 0 0.01 0.02
−17:1D 0 0.02 0 0 0 0.03
−33:36D 0.01 0.02 0 0 0 0.01
−30 −25 −20 −15 −10 −5 −1 1 5 10 15 20
Parentals
Uninjected
dCas9
Cas9enzyme
Cas9RNA
Scrambled
Percentage
0
25
50
75
100

atp1a1a.1:

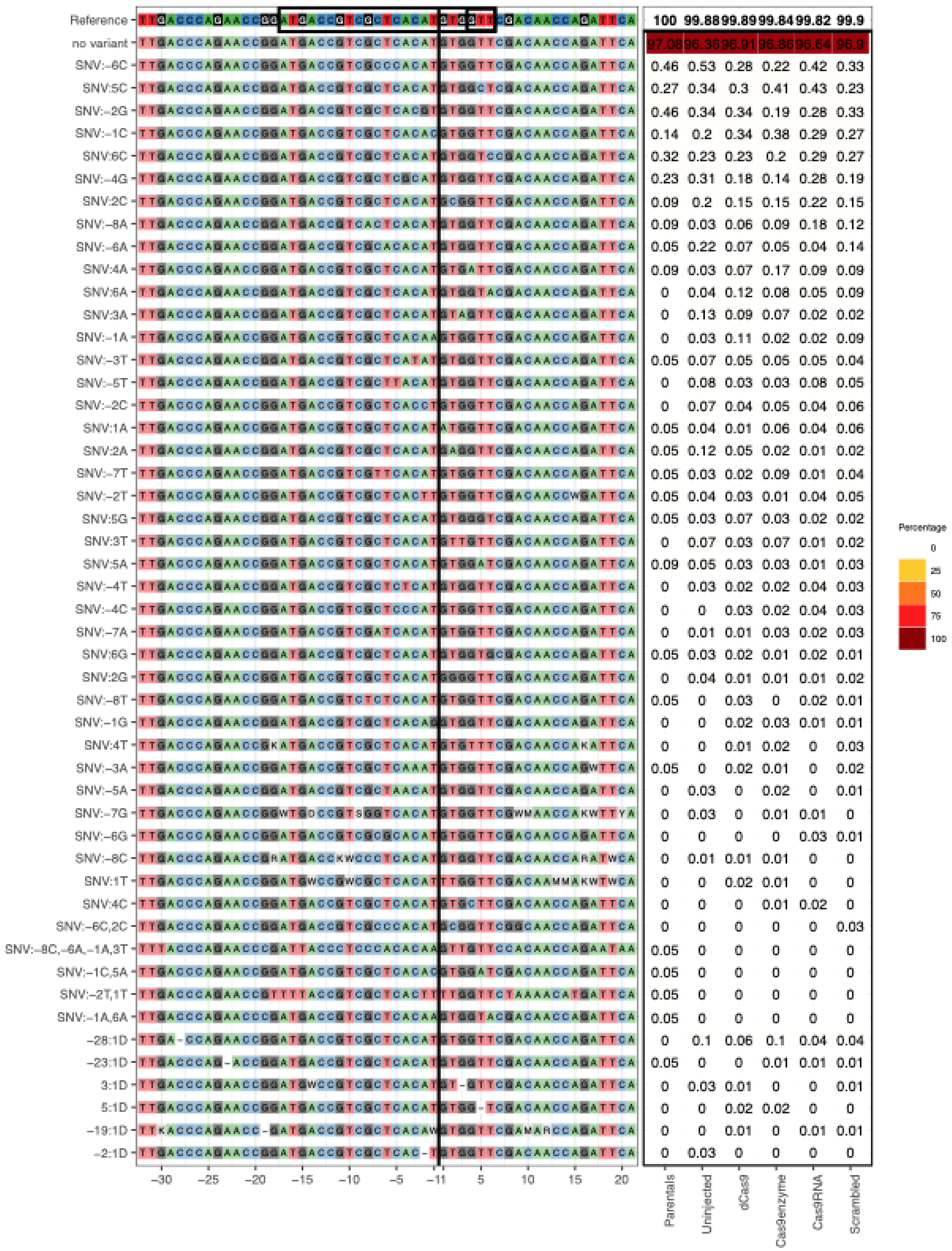

Reference
100 99.88 99.89 99.84 99.82 99.9
no variant
97.08 96.36 96.91 96.86 96.64 96.9
SNV:-6C 0.46 0.53 0.28 0.22 0.42 0.33
SNV:5C 0.27 0.34 0.3 0.41 0.43 0.23
SNV:-2G 0.46 0.34 0.34 0.19 0.28 0.33
SNV:-1C 0.14 0.2 0.34 0.38 0.29 0.27
SNV:6C 0.32 0.23 0.23 0.2 0.29 0.27
SNV:-4G 0.23 0.31 0.18 0.14 0.28 0.19
SNV:2C 0.09 0.2 0.15 0.15 0.22 0.15
SNV:-8A 0.09 0.03 0.06 0.09 0.18 0.12
SNV:-6A 0.05 0.22 0.07 0.05 0.04 0.14
SNV:4A 0.09 0.03 0.07 0.17 0.09 0.09
SNV:6A 0 0.04 0.12 0.08 0.05 0.09
SNV:3A 0 0.13 0.09 0.07 0.02 0.02
SNV:-1A 0 0.03 0.11 0.02 0.02 0.09
SNV:-3T 0.05 0.07 0.05 0.05 0.05 0.04
SNV:-5T 0 0.08 0.03 0.03 0.08 0.05
SNV:-2C 0 0.07 0.04 0.05 0.04 0.06
SNV:1A 0.05 0.04 0.01 0.06 0.04 0.06
SNV:2A 0.05 0.12 0.05 0.02 0.01 0.02
SNV:-7T 0.05 0.03 0.02 0.09 0.01 0.04
SNV:-2T 0.05 0.04 0.03 0.01 0.04 0.05
SNV:5G 0.05 0.03 0.07 0.03 0.02 0.02
SNV:3T 0 0.07 0.03 0.07 0.01 0.02
SNV:5A 0.09 0.05 0.03 0.03 0.01 0.03
SNV:-4T 0 0.03 0.02 0.02 0.04 0.03
SNV:-4C 0 0 0.03 0.02 0.04 0.03
SNV:-7A 0 0.01 0.01 0.03 0.02 0.03
SNV:6G 0.05 0.03 0.02 0.01 0.02 0.01
SNV:2G 0 0.04 0.01 0.01 0.01 0.02
SNV:-8T 0.05 0 0.03 0 0.02 0.01
SNV:-1G 0 0 0.02 0.03 0.01 0.01
SNV:4T 0 0 0.01 0.02 0 0.03
SNV:-3A 0.05 0 0.02 0.01 0 0.02
SNV:-5A 0 0.03 0 0.02 0 0.01
SNV:-7G 0 0.03 0 0.01 0.01 0
SNV:-6G 0 0 0 0 0.03 0.01
SNV:-8C 0 0.01 0.01 0.01 0 0
SNV:1T 0 0 0.02 0.01 0 0
SNV:4C 0 0 0 0.01 0.02 0
SNV:-6C,2C 0 0 0 0 0 0.03
SNV:-8C,-6A,-1A,3T 0.05 0 0 0 0 0
SNV:-1C,5A 0.05 0 0 0 0 0
SNV:-2T,1T 0.05 0 0 0 0 0
SNV:-1A,6A 0.05 0 0 0 0 0
-28:1D 0 0.1 0.06 0.1 0.04 0.04
-23:1D 0.05 0 0 0.01 0.01 0.01
3:1D 0 0.03 0.01 0 0 0.01
5:1D 0 0 0.02 0.02 0 0
-19:1D 0 0 0.01 0 0.01 0.01
-2:1D 0 0.03 0 0 0 0
-30 -25 -20 -15 -10 -5 -1 1 5 10 15 20
Parentals
Uninjected
dCas9
Cas9enzyme
Cas9RNA
Scrambled
Percentage
0
25
50
75
100

atp5md:

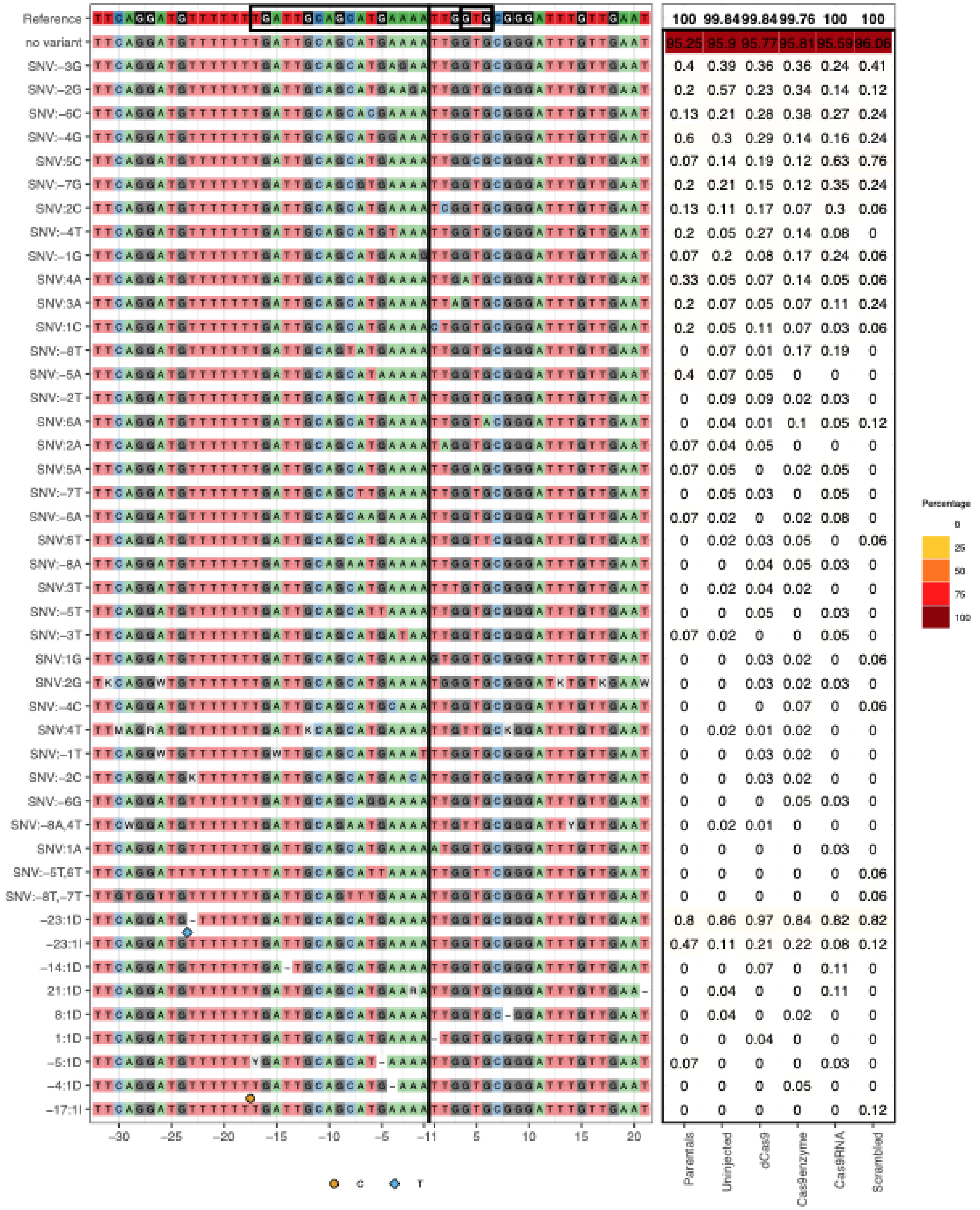
Reference 100 99.84 99.84 99.76 100 100
no variant 95.25 95.9 95.77 95.81 95.59 96.06
SNV:-3G 0.4 0.39 0.36 0.36 0.24 0.41
SNV:-2G 0.2 0.57 0.23 0.34 0.14 0.12
SNV:-6C 0.13 0.21 0.28 0.38 0.27 0.24
SNV:-4G 0.6 0.3 0.29 0.14 0.16 0.24
SNV:5C 0.07 0.14 0.19 0.12 0.63 0.76
SNV:-7G 0.2 0.21 0.15 0.12 0.35 0.24
SNV:2C 0.13 0.11 0.17 0.07 0.3 0.06
SNV:-4T 0.2 0.05 0.27 0.14 0.08 0
SNV:-1G 0.07 0.2 0.08 0.17 0.24 0.06
SNV:4A 0.33 0.05 0.07 0.14 0.05 0.06
SNV:3A 0.2 0.07 0.05 0.07 0.11 0.24
SNV:1C 0.2 0.05 0.11 0.07 0.03 0.06
SNV:-8T 0 0.07 0.01 0.17 0.19 0
SNV:-5A 0.4 0.07 0.05 0 0 0
SNV:-2T 0 0.09 0.09 0.02 0.03 0
SNV:6A 0 0.04 0.01 0.1 0.05 0.12
SNV:2A 0.07 0.04 0.05 0 0 0
SNV:5A 0.07 0.05 0 0.02 0.05 0
SNV:-7T 0 0.05 0.03 0 0.05 0
SNV:-6A 0.07 0.02 0 0.02 0.08 0
SNV:6T 0 0.02 0.03 0.05 0 0.06
SNV:-8A 0 0 0.04 0.05 0.03 0
SNV:3T 0 0.02 0.04 0.02 0 0
SNV:-5T 0 0 0.05 0 0.03 0
SNV:-3T 0.07 0.02 0 0 0.05 0
SNV:1G 0 0 0.03 0.02 0 0.06
SNV:2G 0 0 0.03 0.02 0.03 0
SNV:-4C 0 0 0 0.07 0 0.06
SNV:4T 0 0.02 0.01 0.02 0 0
SNV:-1T 0 0 0.03 0.02 0 0
SNV:-2C 0 0 0.03 0.02 0 0
SNV:-6G 0 0 0 0.05 0.03 0
SNV:-8A,4T 0 0.02 0.01 0 0 0
SNV:1A 0 0 0 0 0.03 0
SNV:-5T,6T 0 0 0 0 0 0.06
SNV:-8T,-7T 0 0 0 0 0 0.06
-23:1D 0.8 0.86 0.97 0.84 0.82 0.82
-23:1I 0.47 0.11 0.21 0.22 0.08 0.12
-14:1D 0 0 0.07 0 0.11 0
21:1D 0 0.04 0 0 0.11 0
8:1D 0 0.04 0 0.02 0 0
1:1D 0 0 0.04 0 0 0
-5:1D 0.07 0 0 0 0.03 0
-4:1D 0 0 0 0.05 0 0
-17:1I 0 0 0 0 0 0.12
Parentals Uninjected dCas9 Cas9enzyme Cas9RNA Scrambled
Percentage 0 25 50 75 100
C T

c3a.1:

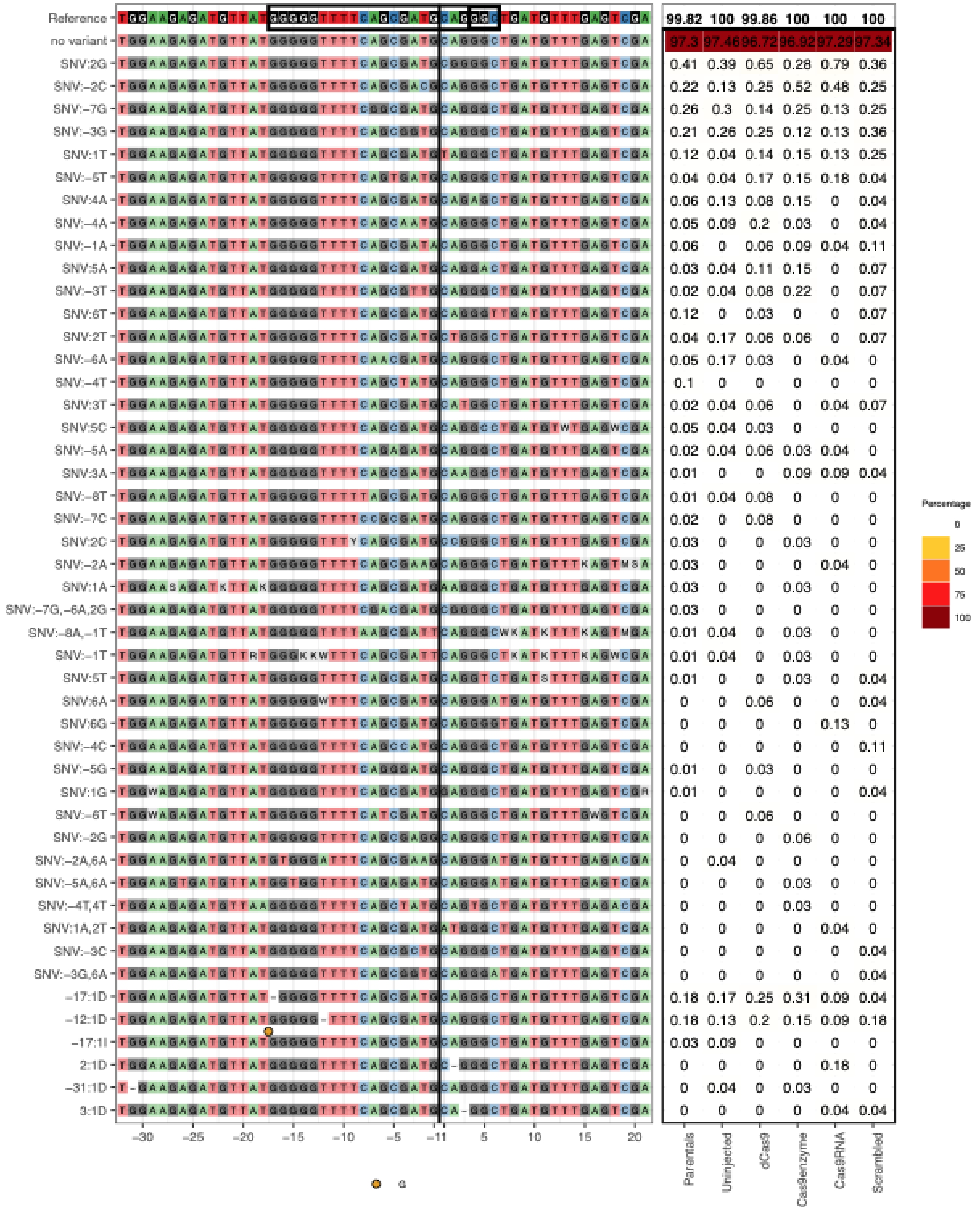
Reference 99.82 100 99.86 100 100 100
no variant 97.3 97.46 96.72 96.92 97.29 97.34
SNV:2G 0.41 0.39 0.65 0.28 0.79 0.36
SNV:−2C 0.22 0.13 0.25 0.52 0.48 0.25
SNV:−7G 0.26 0.3 0.14 0.25 0.13 0.25
SNV:−3G 0.21 0.26 0.25 0.12 0.13 0.36
SNV:1T 0.12 0.04 0.14 0.15 0.13 0.25
SNV:−5T 0.04 0.04 0.17 0.15 0.18 0.04
SNV:4A 0.06 0.13 0.08 0.15 0 0.04
SNV:−4A 0.05 0.09 0.2 0.03 0 0.04
SNV:−1A 0.06 0 0.06 0.09 0.04 0.11
SNV:5A 0.03 0.04 0.11 0.15 0 0.07
SNV:−3T 0.02 0.04 0.08 0.22 0 0.07
SNV:6T 0.12 0 0.03 0 0 0.07
SNV:2T 0.04 0.17 0.06 0.06 0 0.07
SNV:−6A 0.05 0.17 0.03 0 0.04 0
SNV:−4T 0.1 0 0 0 0 0
SNV:3T 0.02 0.04 0.06 0 0.04 0.07
SNV:5C 0.05 0.04 0.03 0 0 0
SNV:−5A 0.02 0.04 0.06 0.03 0.04 0
SNV:3A 0.01 0 0 0.09 0.09 0.04
SNV:−8T 0.01 0.04 0.08 0 0 0
SNV:−7C 0.02 0 0.08 0 0 0
SNV:2C 0.03 0 0 0.03 0 0
SNV:−2A 0.03 0 0 0 0.04 0
SNV:1A 0.03 0 0 0.03 0 0
SNV:−7G,−6A,2G 0.03 0 0 0 0 0
SNV:−8A,−1T 0.01 0.04 0 0.03 0 0
SNV:−1T 0.01 0.04 0 0.03 0 0
SNV:5T 0.01 0 0 0.03 0 0.04
SNV:6A 0 0 0.06 0 0 0.04
SNV:6G 0 0 0 0 0.13 0
SNV:−4C 0 0 0 0 0 0.11
SNV:−5G 0.01 0 0.03 0 0 0
SNV:1G 0.01 0 0 0 0 0.04
SNV:−6T 0 0 0.06 0 0 0
SNV:−2G 0 0 0 0.06 0 0
SNV:−2A,6A 0 0.04 0 0 0 0
SNV:−5A,6A 0 0 0 0.03 0 0
SNV:−4T,4T 0 0 0 0.03 0 0
SNV:1A,2T 0 0 0 0 0.04 0
SNV:−3C 0 0 0 0 0 0.04
SNV:−3G,6A 0 0 0 0 0 0.04
−17:1D 0.18 0.17 0.25 0.31 0.09 0.04
−12:1D 0.18 0.13 0.2 0.15 0.09 0.18
−17:1I 0.03 0.09 0 0 0 0
2:1D 0 0 0 0 0.18 0
−31:1D 0 0.04 0 0.03 0 0
3:1D 0 0 0 0 0.04 0.04
−30 −25 −20 −15 −10 −5 −1 1 5 10 15 20
Parentals
Uninjected
dCas9
Cas9enzyme
Cas9RNA
Scrambled
G
Percentage
0
25
50
75
100

fabp2:

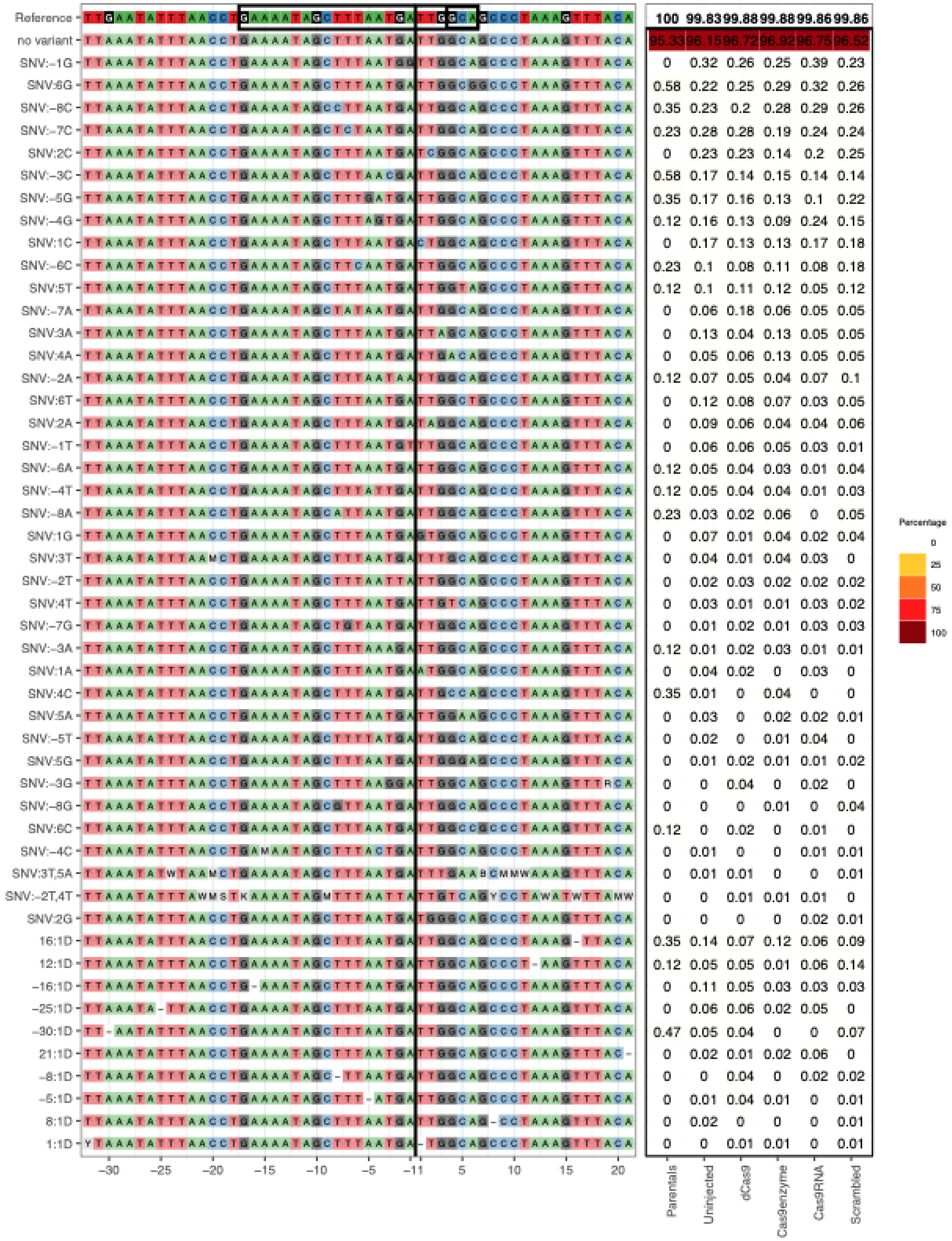
Reference 100 99.83 99.88 99.88 99.86 99.86
no variant 95.33 96.15 96.72 96.92 96.75 96.52
SNV:-1G 0 0.32 0.26 0.25 0.39 0.23
SNV:6G 0.58 0.22 0.25 0.29 0.32 0.26
SNV:-8C 0.35 0.23 0.2 0.28 0.29 0.26
SNV:-7C 0.23 0.28 0.28 0.19 0.24 0.24
SNV:2C 0 0.23 0.23 0.14 0.2 0.25
SNV:-3C 0.58 0.17 0.14 0.15 0.14 0.14
SNV:-5G 0.35 0.17 0.16 0.13 0.1 0.22
SNV:-4G 0.12 0.16 0.13 0.09 0.24 0.15
SNV:1C 0 0.17 0.13 0.13 0.17 0.18
SNV:-6C 0.23 0.1 0.08 0.11 0.08 0.18
SNV:5T 0.12 0.1 0.11 0.12 0.05 0.12
SNV:-7A 0 0.06 0.18 0.06 0.05 0.05
SNV:3A 0 0.13 0.04 0.13 0.05 0.05
SNV:4A 0 0.05 0.06 0.13 0.05 0.05
SNV:-2A 0.12 0.07 0.05 0.04 0.07 0.1
SNV:6T 0 0.12 0.08 0.07 0.03 0.05
SNV:2A 0 0.09 0.06 0.04 0.04 0.06
SNV:-1T 0 0.06 0.06 0.05 0.03 0.01
SNV:-6A 0.12 0.05 0.04 0.03 0.01 0.04
SNV:-4T 0.12 0.05 0.04 0.04 0.01 0.03
SNV:-8A 0.23 0.03 0.02 0.06 0 0.05
SNV:1G 0 0.07 0.01 0.04 0.02 0.04
SNV:3T 0 0.04 0.01 0.04 0.03 0
SNV:-2T 0 0.02 0.03 0.02 0.02 0.02
SNV:4T 0 0.03 0.01 0.01 0.03 0.02
SNV:-7G 0 0.01 0.02 0.01 0.03 0.03
SNV:-3A 0.12 0.01 0.02 0.03 0.01 0.01
SNV:1A 0 0.04 0.02 0 0.03 0
SNV:4C 0.35 0.01 0 0.04 0 0
SNV:5A 0 0.03 0 0.02 0.02 0.01
SNV:-5T 0 0.02 0 0.01 0.04 0
SNV:5G 0 0.01 0.02 0.01 0.01 0.02
SNV:-3G 0 0 0.04 0 0.02 0
SNV:-8G 0 0 0 0.01 0 0.04
SNV:6C 0.12 0 0.02 0 0.01 0
SNV:-4C 0 0.01 0 0 0.01 0.01
SNV:3T,5A 0 0.01 0.01 0 0 0.01
SNV:-2T,4T 0 0 0.01 0.01 0.01 0
SNV:2G 0 0 0 0 0.02 0.01
16:1D 0.35 0.14 0.07 0.12 0.06 0.09
12:1D 0.12 0.05 0.05 0.01 0.06 0.14
-16:1D 0 0.11 0.05 0.03 0.03 0.03
-25:1D 0 0.06 0.06 0.02 0.05 0
-30:1D 0.47 0.05 0.04 0 0 0.07
21:1D 0 0.02 0.01 0.02 0.06 0
-8:1D 0 0 0.04 0 0.02 0.02
-5:1D 0 0.01 0.04 0.01 0 0.01
8:1D 0 0.02 0 0 0 0.01
1:1D 0 0 0.01 0.01 0 0.01
-30 -25 -20 -15 -10 -5 -1 5 10 15 20
Parentals
Uninjected
dCas9
Cas9enzyme
Cas9RNA
Scrambled
Percentage
0
25
50
75
100

fga:

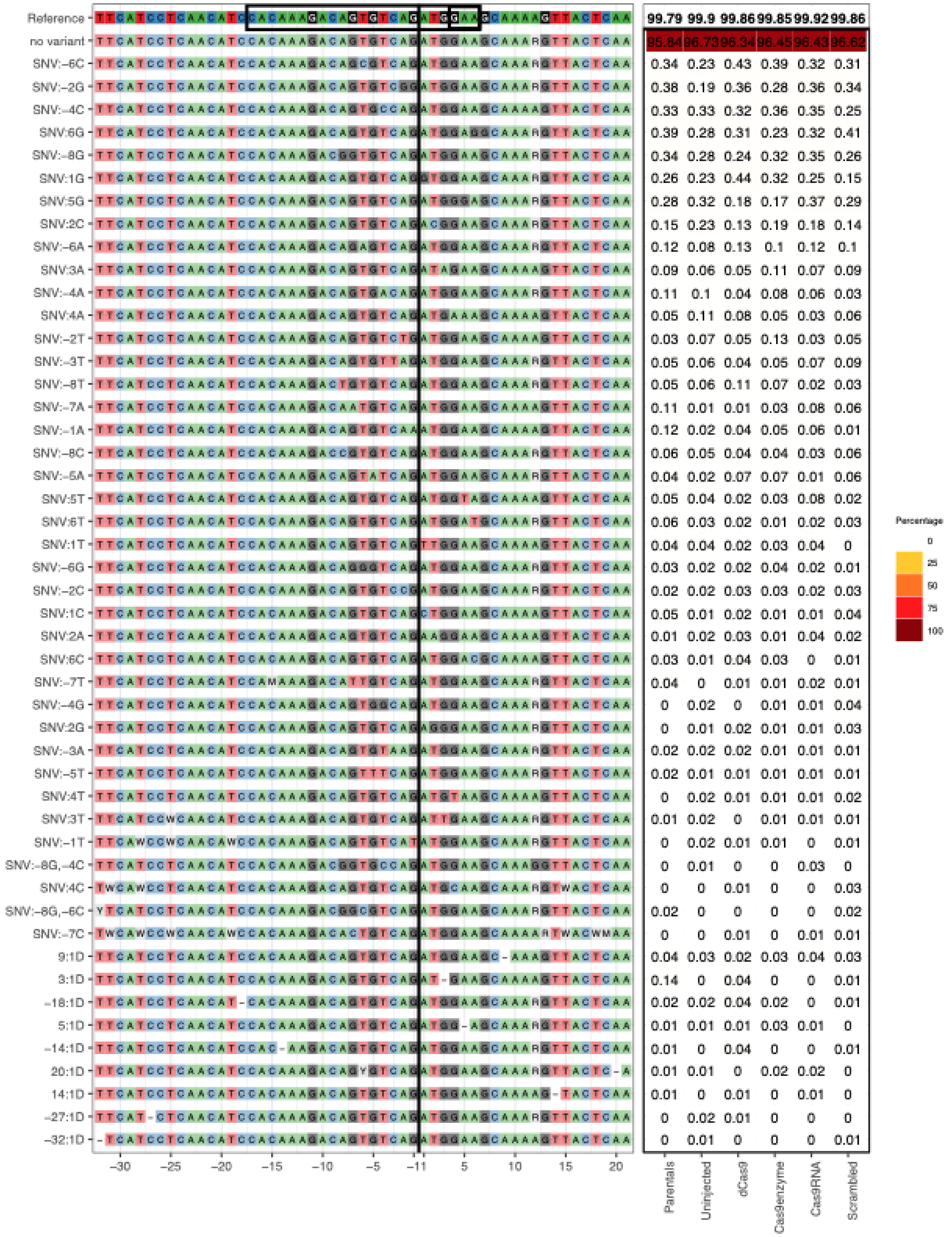
Reference
no variant
Parentals
Uninjected
dCas9
Cas9enzyme
Cas9RNA
Scrambled
Percentage

fgg:

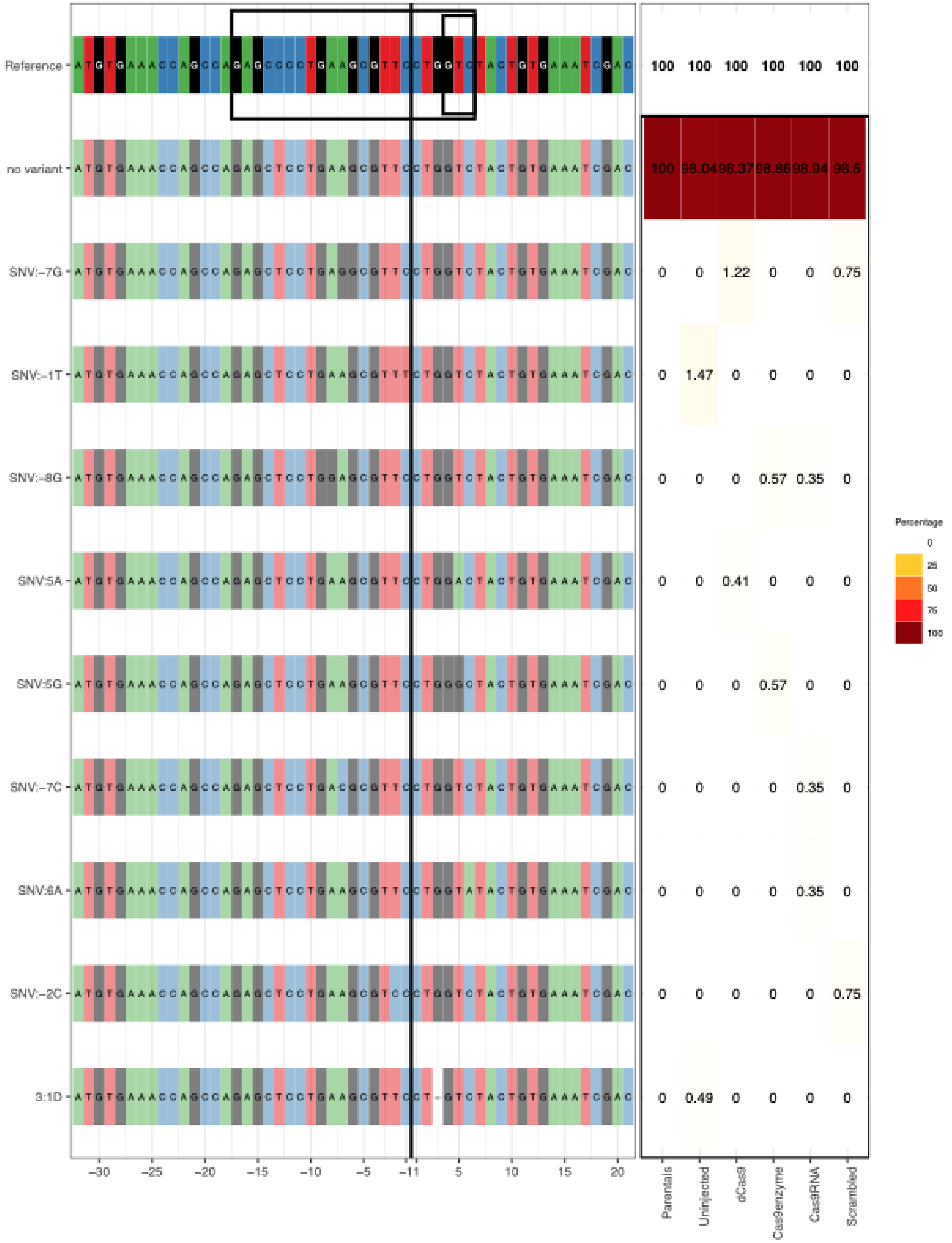
Reference
no variant
SNV:-7G
SNV:-1T
SNV:-8G
SNV:5A
SNV:5G
SNV:-7C
SNV:6A
SNV:-2C
3:1D
100 100 100 100 100 100
100 98.04 98.37 98.86 98.94 98.5
0 0 1.22 0 0 0.75
0 1.47 0 0 0 0
0 0 0 0.57 0.35 0
0 0 0.41 0 0 0
0 0 0 0.57 0 0
0 0 0 0 0.35 0
0 0 0 0 0.35 0
0 0 0 0 0 0.75
0 0.49 0 0 0 0
−30 −25 −20 −15 −10 −5 −1 1 5 10 15 20
Parentals
Uninjected
dCas9
Cas9enzyme
Cas9RNA
Scrambled
Percentage
0
25
50
75
100

galnt7:

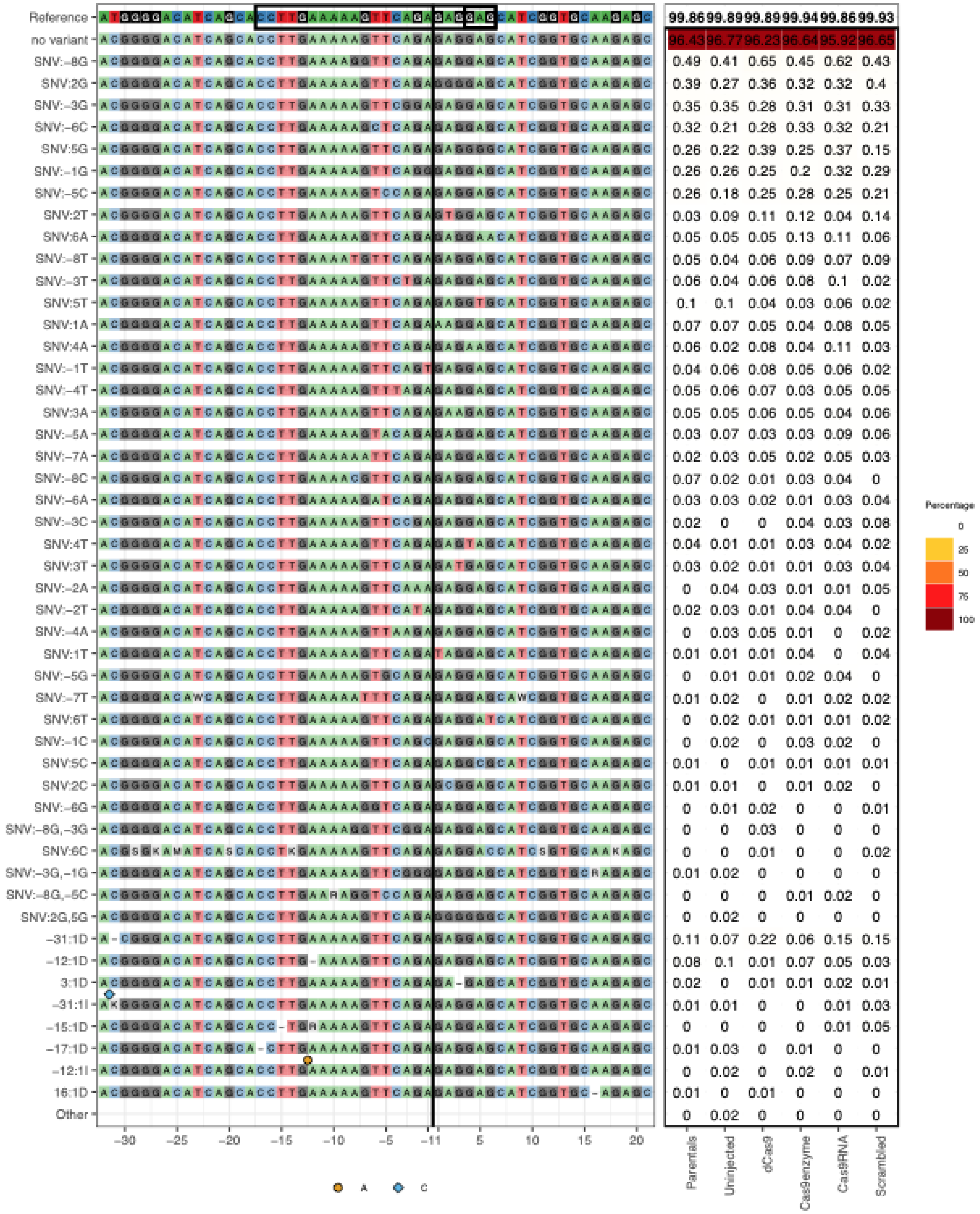
Reference
99.86 99.89 99.89 99.94 99.86 99.93
no variant 96.43 96.77 96.23 96.64 95.92 96.65
SNV:-8G 0.49 0.41 0.65 0.45 0.62 0.43
SNV:2G 0.39 0.27 0.36 0.32 0.32 0.4
SNV:-3G 0.35 0.35 0.28 0.31 0.31 0.33
SNV:-6C 0.32 0.21 0.28 0.33 0.32 0.21
SNV:5G 0.26 0.22 0.39 0.25 0.37 0.15
SNV:-1G 0.26 0.26 0.25 0.2 0.32 0.29
SNV:-5C 0.26 0.18 0.25 0.28 0.25 0.21
SNV:2T 0.03 0.09 0.11 0.12 0.04 0.14
SNV:6A 0.05 0.05 0.05 0.13 0.11 0.06
SNV:-8T 0.05 0.04 0.06 0.09 0.07 0.09
SNV:-3T 0.06 0.04 0.06 0.08 0.1 0.02
SNV:5T 0.1 0.1 0.04 0.03 0.06 0.02
SNV:1A 0.07 0.07 0.05 0.04 0.08 0.05
SNV:4A 0.06 0.02 0.08 0.04 0.11 0.03
SNV:-1T 0.04 0.06 0.08 0.05 0.06 0.02
SNV:-4T 0.05 0.06 0.07 0.03 0.05 0.05
SNV:3A 0.05 0.05 0.06 0.05 0.04 0.06
SNV:-5A 0.03 0.07 0.03 0.03 0.09 0.06
SNV:-7A 0.02 0.03 0.05 0.02 0.05 0.03
SNV:-8C 0.07 0.02 0.01 0.03 0.04 0
SNV:-6A 0.03 0.03 0.02 0.01 0.03 0.04
SNV:-3C 0.02 0 0 0.04 0.03 0.08
SNV:4T 0.04 0.01 0.01 0.03 0.04 0.02
SNV:3T 0.03 0.02 0.01 0.01 0.03 0.04
SNV:-2A 0 0.04 0.03 0.01 0.01 0.05
SNV:-2T 0.02 0.03 0.01 0.04 0.04 0
SNV:-4A 0 0.03 0.05 0.01 0 0.02
SNV:1T 0.01 0.01 0.01 0.04 0 0.04
SNV:-5G 0 0.01 0.01 0.02 0.04 0
SNV:-7T 0.01 0.02 0 0.01 0.02 0.02
SNV:6T 0 0.02 0.01 0.01 0.01 0.02
SNV:-1C 0 0.02 0 0.03 0.02 0
SNV:5C 0.01 0 0.01 0.01 0.01 0.01
SNV:2C 0.01 0.01 0 0.01 0.02 0
SNV:-6G 0 0.01 0.02 0 0 0.01
SNV:-8G,-3G 0 0 0.03 0 0 0
SNV:6C 0 0 0.01 0 0 0.02
SNV:-3G,-1G 0.01 0.02 0 0 0 0
SNV:-8G,-5C 0 0 0 0.01 0.02 0
SNV:2G,5G 0 0.02 0 0 0 0
-31:1D 0.11 0.07 0.22 0.06 0.15 0.15
-12:1D 0.08 0.1 0.01 0.07 0.05 0.03
3:1D 0.02 0 0.01 0.01 0.02 0.01
-31:1I 0.01 0.01 0 0 0.01 0.03
-15:1D 0 0 0 0 0.01 0.05
-17:1D 0.01 0.03 0 0.01 0 0
-12:1I 0 0.02 0 0.02 0 0.01
16:1D 0.01 0 0.01 0 0 0
Other 0 0.02 0 0 0 0
-30 -25 -20 -15 -10 -5 -1 1 5 10 15 20
Parentals Uninjected dCas9 Cas9enzyme Cas9RNA Scrambled
A C
Percentage
0 25 50 75 100

ggact.2:

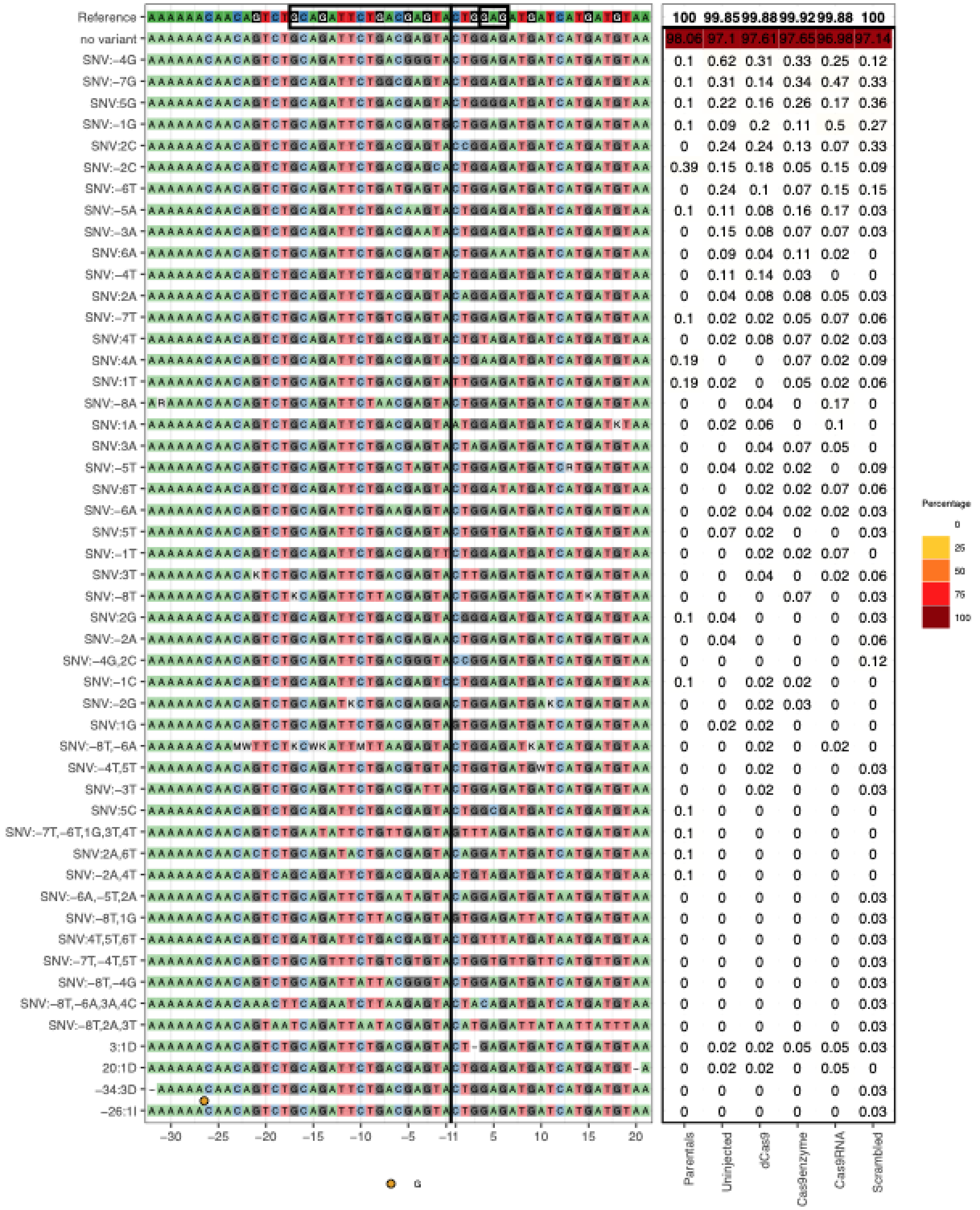
Reference 100 99.85 99.88 99.92 99.88 100
no variant 98.06 97.1 97.61 97.65 96.98 97.14
SNV:-4G 0.1 0.62 0.31 0.33 0.25 0.12
SNV:-7G 0.1 0.31 0.14 0.34 0.47 0.33
SNV:5G 0.1 0.22 0.16 0.26 0.17 0.36
SNV:-1G 0.1 0.09 0.2 0.11 0.5 0.27
SNV:2C 0 0.24 0.24 0.13 0.07 0.33
SNV:-2C 0.39 0.15 0.18 0.05 0.15 0.09
SNV:-6T 0 0.24 0.1 0.07 0.15 0.15
SNV:-5A 0.1 0.11 0.08 0.16 0.17 0.03
SNV:-3A 0 0.15 0.08 0.07 0.07 0.03
SNV:6A 0 0.09 0.04 0.11 0.02 0
SNV:-4T 0 0.11 0.14 0.03 0 0
SNV:2A 0 0.04 0.08 0.08 0.05 0.03
SNV:-7T 0.1 0.02 0.02 0.05 0.07 0.06
SNV:4T 0 0.02 0.08 0.07 0.02 0.03
SNV:4A 0.19 0 0 0.07 0.02 0.09
SNV:1T 0.19 0.02 0 0.05 0.02 0.06
SNV:-8A 0 0 0.04 0 0.17 0
SNV:1A 0 0.02 0.06 0 0.1 0
SNV:3A 0 0 0.04 0.07 0.05 0
SNV:-5T 0 0.04 0.02 0.02 0 0.09
SNV:6T 0 0 0.02 0.02 0.07 0.06
SNV:-6A 0 0.02 0.04 0.02 0.02 0.03
SNV:5T 0 0.07 0.02 0 0 0.03
SNV:-1T 0 0 0.02 0.02 0.07 0
SNV:3T 0 0 0.04 0 0.02 0.06
SNV:-8T 0 0 0 0.07 0 0.03
SNV:2G 0.1 0.04 0 0 0 0.03
SNV:-2A 0 0.04 0 0 0 0.06
SNV:-4G,2C 0 0 0 0 0 0.12
SNV:-1C 0.1 0 0.02 0.02 0 0
SNV:-2G 0 0 0.02 0.03 0 0
SNV:1G 0 0.02 0.02 0 0 0
SNV:-8T,-6A 0 0 0.02 0 0.02 0
SNV:-4T,5T 0 0 0.02 0 0 0.03
SNV:-3T 0 0 0.02 0 0 0.03
SNV:5C 0.1 0 0 0 0 0
SNV:-7T,-6T,1G,3T,4T 0.1 0 0 0 0 0
SNV:2A,6T 0.1 0 0 0 0 0
SNV:-2A,4T 0.1 0 0 0 0 0
SNV:-6A,-5T,2A 0 0 0 0 0 0.03
SNV:-8T,1G 0 0 0 0 0 0.03
SNV:4T,5T,6T 0 0 0 0 0 0.03
SNV:-7T,-4T,5T 0 0 0 0 0 0.03
SNV:-8T,-4G 0 0 0 0 0 0.03
SNV:-8T,-6A,3A,4C 0 0 0 0 0 0.03
SNV:-8T,2A,3T 0 0 0 0 0 0.03
3:1D 0 0.02 0.02 0.05 0.05 0.03
20:1D 0 0.02 0.02 0 0.05 0
-34:3D 0 0 0 0 0 0.03
-26:1I 0 0 0 0 0 0.03
-30 -25 -20 -15 -10 -5 -1 1 5 10 15 20
Parentals
Uninjected
dCas9
Cas9enzyme
Cas9RNA
Scrambled
Percentage
0
25
50
75
100
G

il15:

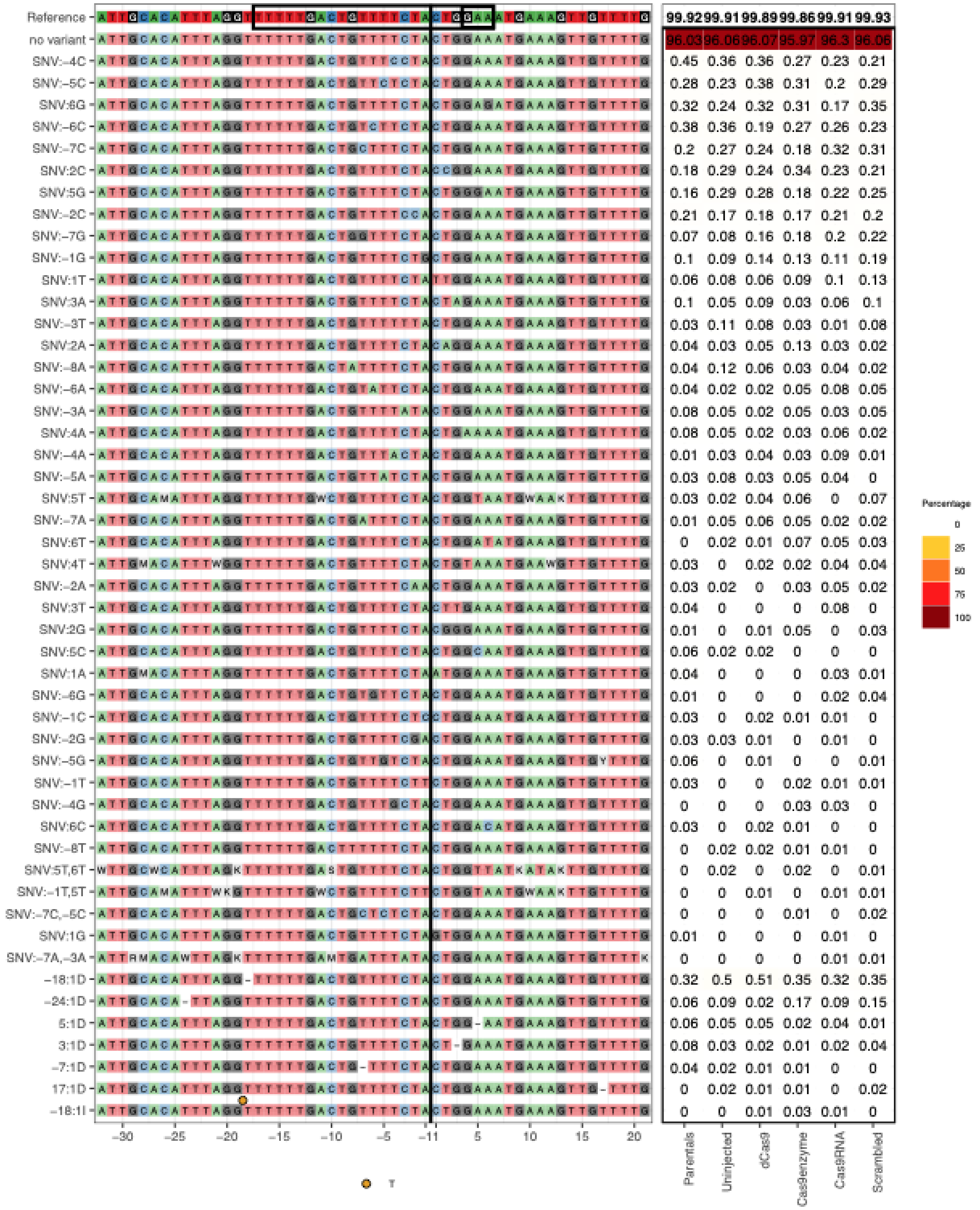
Reference
no variant
Parentals
Uninjected
dCas9
Cas9enzyme
Cas9RNA
Scrambled
Percentage
0
25
50
75
100

knop1:

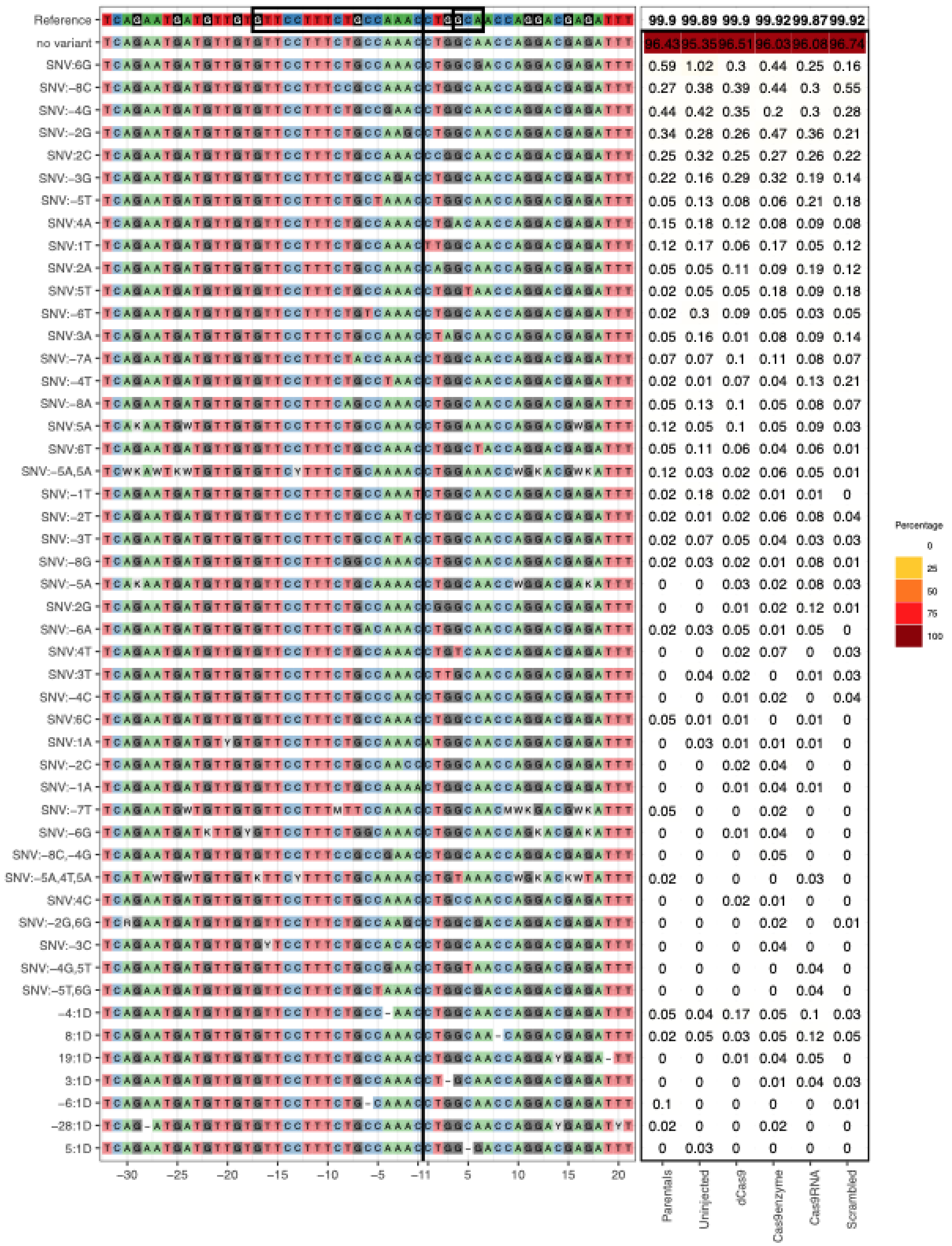
Reference
no variant
Parentals
Uninjected
dCas9
Cas9enzyme
Cas9RNA
Scrambled
Percentage
0
25
50
75
100
−30
−25
−20
−15
−10
−5
−1|1
5
10
15
20

lamp1a:

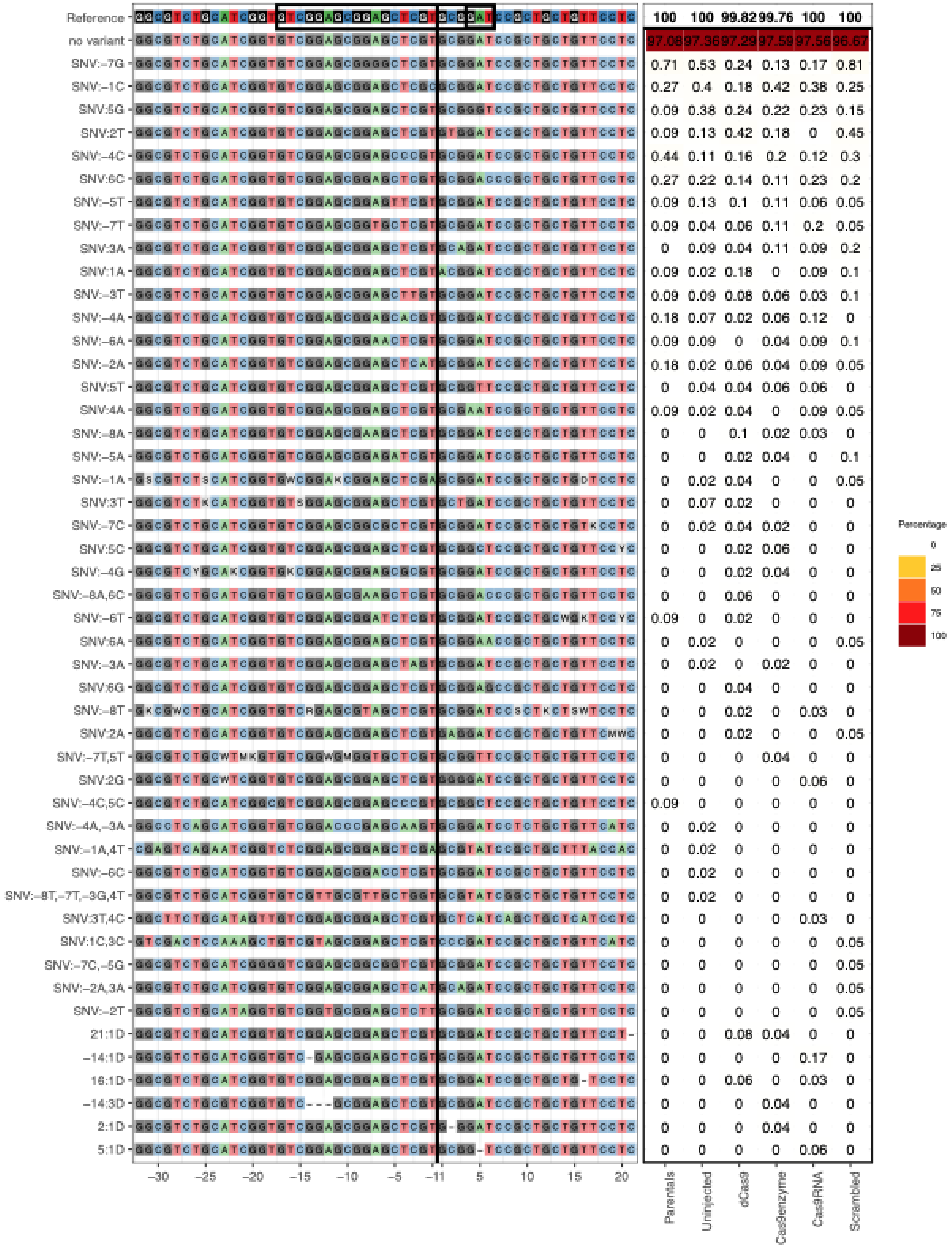
Reference
no variant
SNV:-7G
SNV:-1C
SNV:5G
SNV:2T
SNV:-4C
SNV:6C
SNV:-5T
SNV:-7T
SNV:3A
SNV:1A
SNV:-3T
SNV:-4A
SNV:-6A
SNV:-2A
SNV:5T
SNV:4A
SNV:-8A
SNV:-5A
SNV:-1A
SNV:3T
SNV:-7C
SNV:5C
SNV:-4G
SNV:-8A,6C
SNV:-6T
SNV:6A
SNV:-3A
SNV:6G
SNV:-8T
SNV:2A
SNV:-7T,5T
SNV:2G
SNV:-4C,5C
SNV:-4A,-3A
SNV:-1A,4T
SNV:-6C
SNV:-8T,-7T,-3G,4T
SNV:3T,4C
SNV:1C,3C
SNV:-7C,-5G
SNV:-2A,3A
SNV:-2T
21:1D
-14:1D
16:1D
-14:3D
2:1D
5:1D
-30
-25
-20
-15
-10
-5
-1
5
10
15
20
Parentals
Uninjected
dCas9
Cas9enzyme
Cas9RNA
Scrambled
Percentage
0
25
50
75
100

nansa:

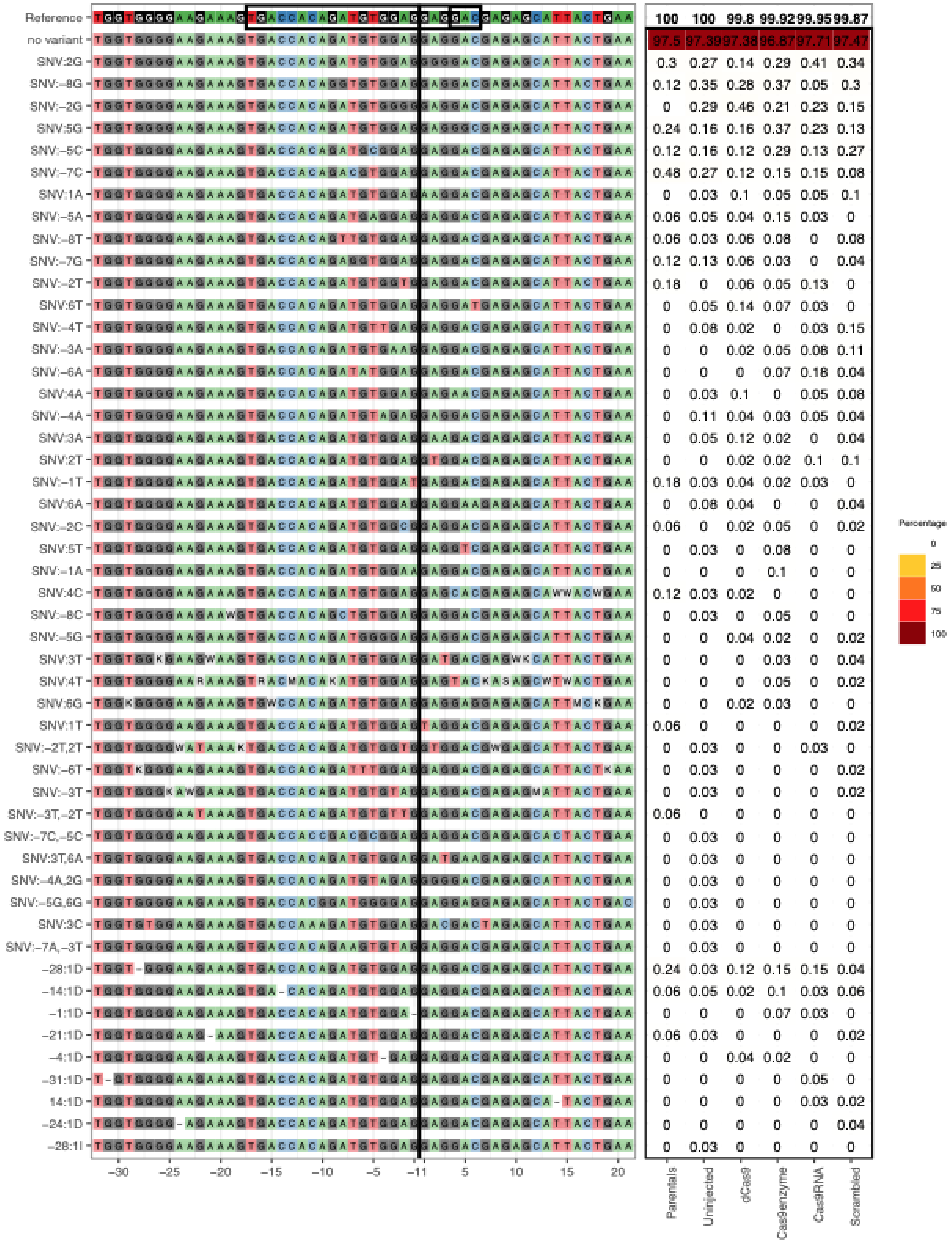
Reference 100 100 99.8 99.92 99.95 99.87
no variant 97.5 97.39 97.38 96.87 97.71 97.47
SNV:2G 0.3 0.27 0.14 0.29 0.41 0.34
SNV:–8G 0.12 0.35 0.28 0.37 0.05 0.3
SNV:–2G 0 0.29 0.46 0.21 0.23 0.15
SNV:5G 0.24 0.16 0.16 0.37 0.23 0.13
SNV:–5C 0.12 0.16 0.12 0.29 0.13 0.27
SNV:–7C 0.48 0.27 0.12 0.15 0.15 0.08
SNV:1A 0 0.03 0.1 0.05 0.05 0.1
SNV:–5A 0.06 0.05 0.04 0.15 0.03 0
SNV:–8T 0.06 0.03 0.06 0.08 0 0.08
SNV:–7G 0.12 0.13 0.06 0.03 0 0.04
SNV:–2T 0.18 0 0.06 0.05 0.13 0
SNV:6T 0 0.05 0.14 0.07 0.03 0
SNV:–4T 0 0.08 0.02 0 0.03 0.15
SNV:–3A 0 0 0.02 0.05 0.08 0.11
SNV:–6A 0 0 0 0.07 0.18 0.04
SNV:4A 0 0.03 0.1 0 0.05 0.08
SNV:–4A 0 0.11 0.04 0.03 0.05 0.04
SNV:3A 0 0.05 0.12 0.02 0 0.04
SNV:2T 0 0 0.02 0.02 0.1 0.1
SNV:–1T 0.18 0.03 0.04 0.02 0.03 0
SNV:6A 0 0.08 0.04 0 0 0.04
SNV:–2C 0.06 0 0.02 0.05 0 0.02
SNV:5T 0 0.03 0 0.08 0 0
SNV:–1A 0 0 0 0.1 0 0
SNV:4C 0.12 0.03 0.02 0 0 0
SNV:–8C 0 0.03 0 0.05 0 0
SNV:–5G 0 0 0.04 0.02 0 0.02
SNV:3T 0 0 0 0.03 0 0.04
SNV:4T 0 0 0 0.05 0 0.02
SNV:6G 0 0 0.02 0.03 0 0
SNV:1T 0.06 0 0 0 0 0.02
SNV:–2T,2T 0 0.03 0 0 0.03 0
SNV:–6T 0 0.03 0 0 0 0.02
SNV:–3T 0 0.03 0 0 0 0.02
SNV:–3T,–2T 0.06 0 0 0 0 0
SNV:–7C,–5C 0 0.03 0 0 0 0
SNV:3T,6A 0 0.03 0 0 0 0
SNV:–4A,2G 0 0.03 0 0 0 0
SNV:–5G,6G 0 0.03 0 0 0 0
SNV:3C 0 0.03 0 0 0 0
SNV:–7A,–3T 0 0.03 0 0 0 0
–28:1D 0.24 0.03 0.12 0.15 0.15 0.04
–14:1D 0.06 0.05 0.02 0.1 0.03 0.06
–1:1D 0 0 0 0.07 0.03 0
–21:1D 0.06 0.03 0 0 0 0.02
–4:1D 0 0 0.04 0.02 0 0
–31:1D 0 0 0 0 0.05 0
14:1D 0 0 0 0 0.03 0.02
–24:1D 0 0 0 0 0 0.04
–28:1I 0 0.03 0 0 0 0
–30 –25 –20 –15 –10 –5 –1 1 5 10 15 20
Parentals
Uninjected
dCas9
Cas9enzyme
Cas9RNA
Scrambled
Percentage
0
25
50
75
100

si:dkey-9i23.15:

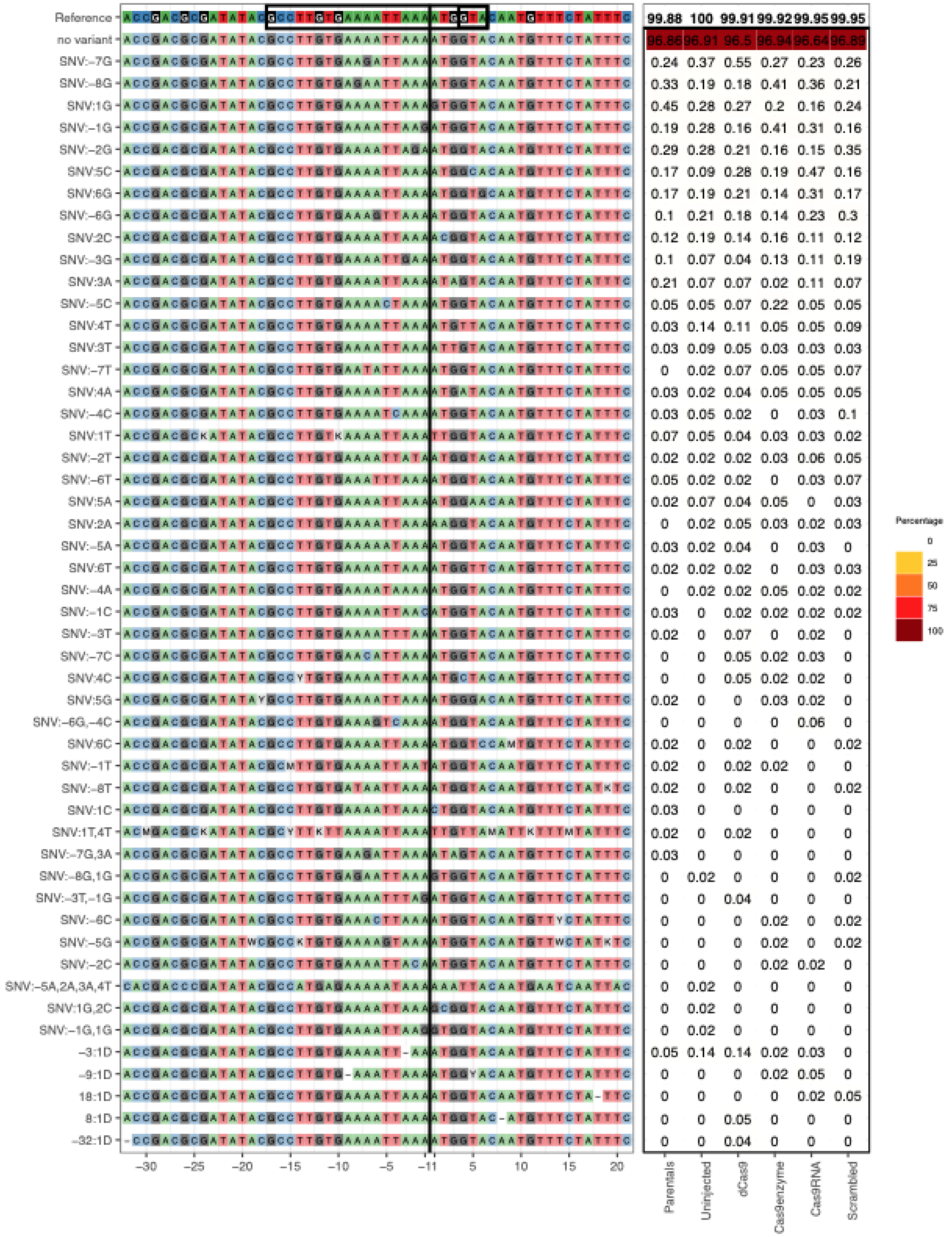

sich1073220a20:

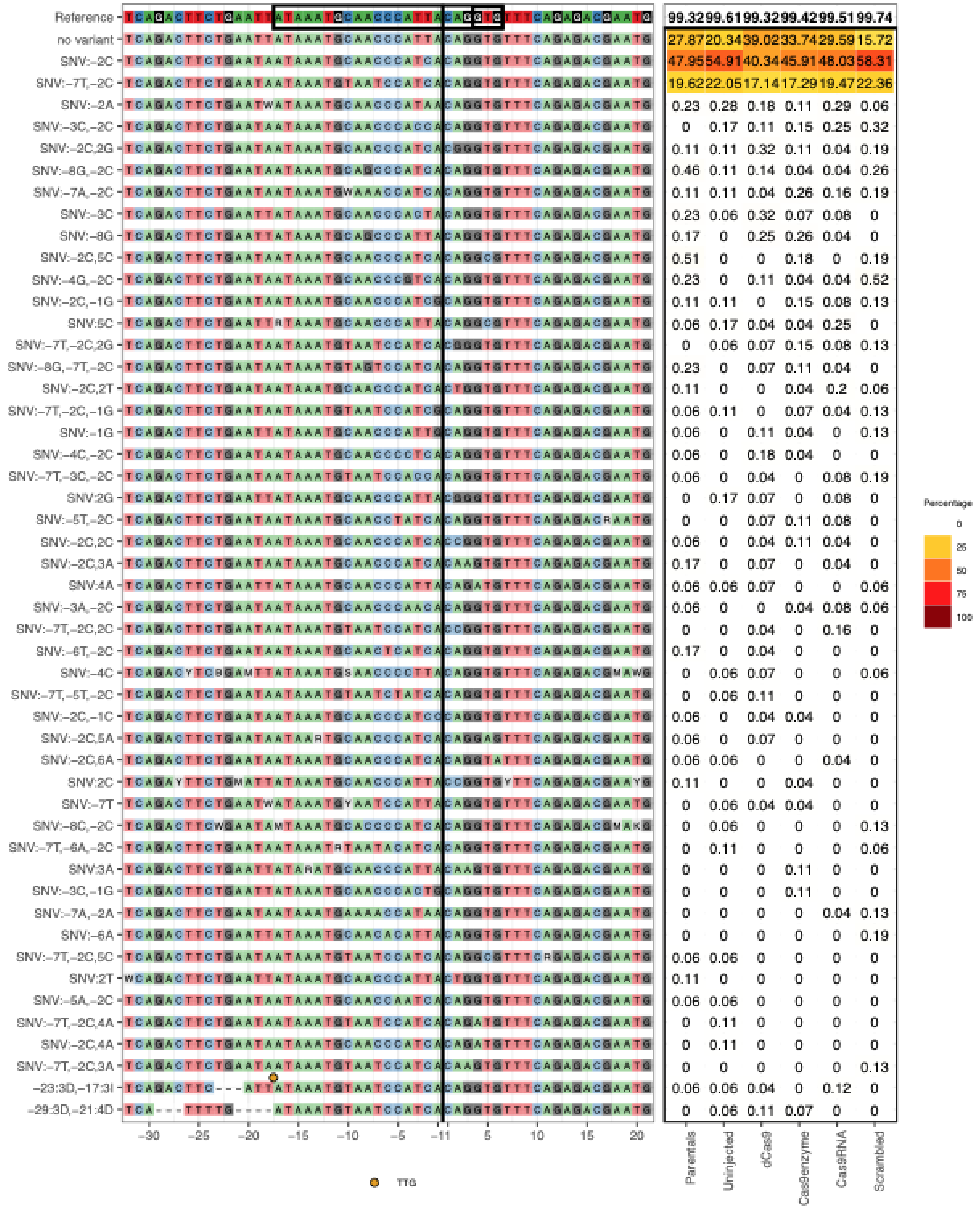

slc25a4:

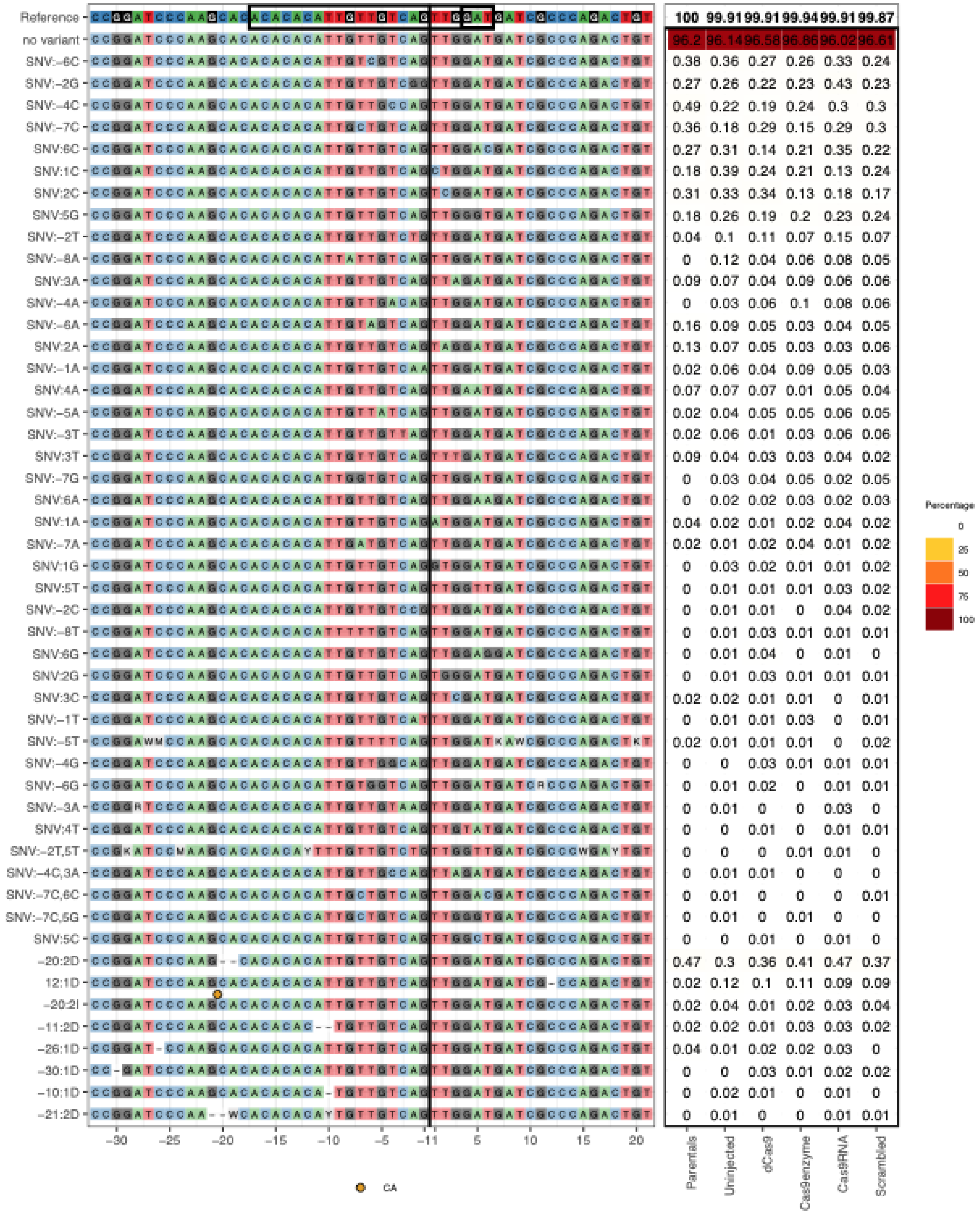
Reference 100 99.91 99.91 99.94 99.91 99.87
no variant 96.2 96.14 96.58 96.86 96.02 96.61
SNV:-6C 0.38 0.36 0.27 0.26 0.33 0.24
SNV:-2G 0.27 0.26 0.22 0.23 0.43 0.23
SNV:-4C 0.49 0.22 0.19 0.24 0.3 0.3
SNV:-7C 0.36 0.18 0.29 0.15 0.29 0.3
SNV:6C 0.27 0.31 0.14 0.21 0.35 0.22
SNV:1C 0.18 0.39 0.24 0.21 0.13 0.24
SNV:2C 0.31 0.33 0.34 0.13 0.18 0.17
SNV:5G 0.18 0.26 0.19 0.2 0.23 0.24
SNV:-2T 0.04 0.1 0.11 0.07 0.15 0.07
SNV:-8A 0 0.12 0.04 0.06 0.08 0.05
SNV:3A 0.09 0.07 0.04 0.09 0.06 0.06
SNV:-4A 0 0.03 0.06 0.1 0.08 0.06
SNV:-6A 0.16 0.09 0.05 0.03 0.04 0.05
SNV:2A 0.13 0.07 0.05 0.03 0.03 0.06
SNV:-1A 0.02 0.06 0.04 0.09 0.05 0.03
SNV:4A 0.07 0.07 0.07 0.01 0.05 0.04
SNV:-5A 0.02 0.04 0.05 0.05 0.06 0.05
SNV:-3T 0.02 0.06 0.01 0.03 0.06 0.06
SNV:3T 0.09 0.04 0.03 0.03 0.04 0.02
SNV:-7G 0 0.03 0.04 0.05 0.02 0.05
SNV:6A 0 0.02 0.02 0.03 0.02 0.03
SNV:1A 0.04 0.02 0.01 0.02 0.04 0.02
SNV:-7A 0.02 0.01 0.02 0.04 0.01 0.02
SNV:1G 0 0.03 0.02 0.01 0.01 0.02
SNV:5T 0 0.01 0.01 0.01 0.03 0.02
SNV:-2C 0 0.01 0.01 0 0.04 0.02
SNV:-8T 0 0.01 0.03 0.01 0.01 0.01
SNV:6G 0 0.01 0.04 0 0.01 0
SNV:2G 0 0.01 0.03 0.01 0.01 0.01
SNV:3C 0.02 0.02 0.01 0.01 0 0.01
SNV:-1T 0 0.01 0.01 0.03 0 0.01
SNV:-5T 0.02 0.01 0.01 0.01 0 0.02
SNV:-4G 0 0 0.03 0.01 0.01 0.01
SNV:-6G 0 0.01 0.02 0 0.01 0.01
SNV:-3A 0 0.01 0 0 0.03 0
SNV:4T 0 0 0.01 0 0.01 0.01
SNV:-2T,5T 0 0 0 0.01 0.01 0
SNV:-4C,3A 0 0.01 0.01 0 0 0
SNV:-7C,6C 0 0.01 0 0 0 0.01
SNV:-7C,5G 0 0.01 0 0.01 0 0
SNV:5C 0 0 0.01 0 0.01 0
-20:2D 0.47 0.3 0.36 0.41 0.47 0.37
12:1D 0.02 0.12 0.1 0.11 0.09 0.09
-20:2I 0.02 0.04 0.01 0.02 0.03 0.04
-11:2D 0.02 0.02 0.01 0.03 0.03 0.02
-26:1D 0.04 0.01 0.02 0.02 0.03 0
-30:1D 0 0 0.03 0.01 0.02 0.02
-10:1D 0 0.02 0.01 0 0.01 0
-21:2D 0 0.01 0 0 0.01 0.01
-30 -25 -20 -15 -10 -5 -1 1 5 10 15 20
Parentals
Uninjected
dCas9
Cas9enzyme
Cas9RNA
Scrambled
CA
Percentage
0
25
50
75
100

spry2:

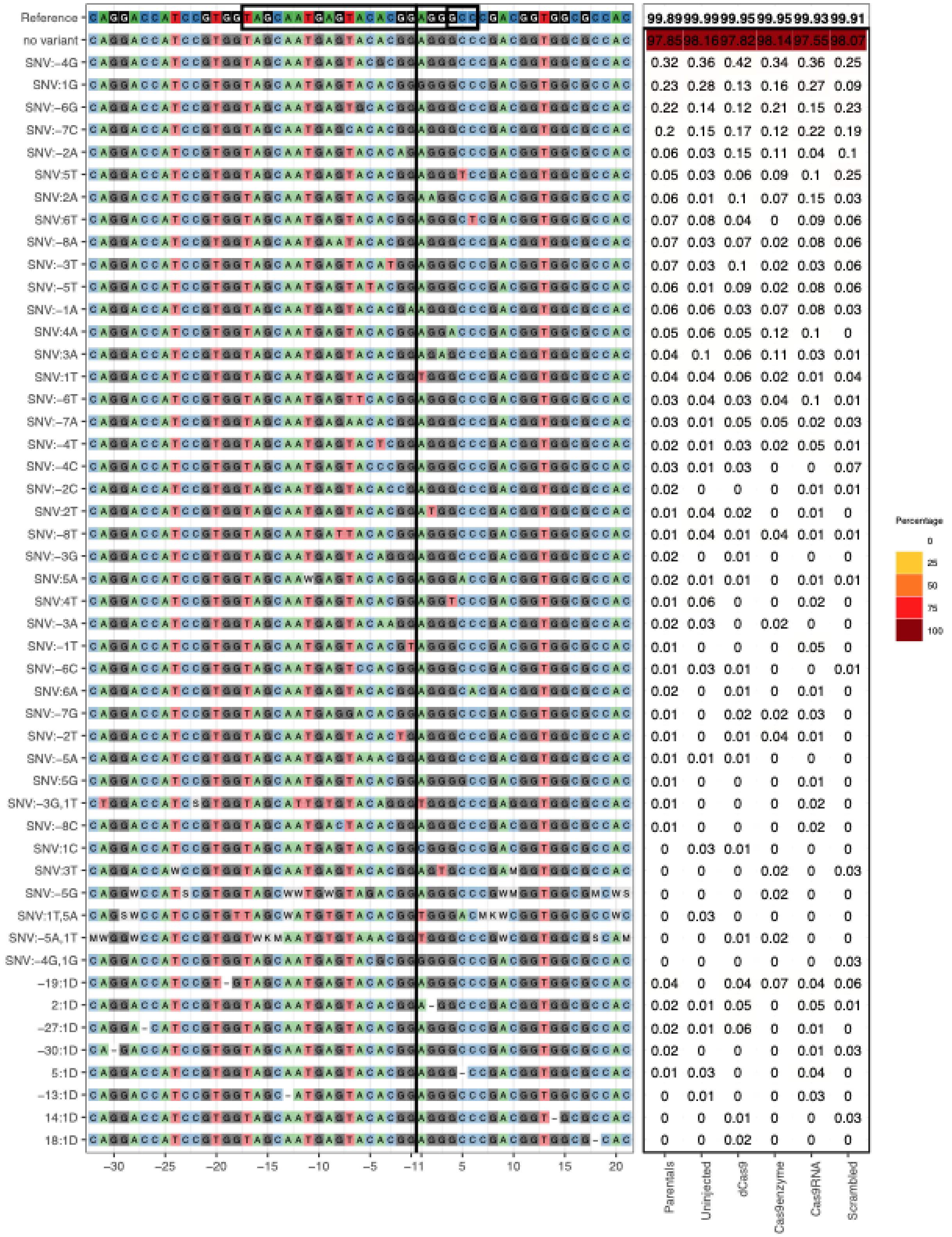
Reference 99.89 99.99 99.95 99.95 99.93 99.91
no variant 97.85 98.16 97.82 98.14 97.55 98.07
SNV:-4G 0.32 0.36 0.42 0.34 0.36 0.25
SNV:1G 0.23 0.28 0.13 0.16 0.27 0.09
SNV:-6G 0.22 0.14 0.12 0.21 0.15 0.23
SNV:-7C 0.2 0.15 0.17 0.12 0.22 0.19
SNV:-2A 0.06 0.03 0.15 0.11 0.04 0.1
SNV:5T 0.05 0.03 0.06 0.09 0.1 0.25
SNV:2A 0.06 0.01 0.1 0.07 0.15 0.03
SNV:6T 0.07 0.08 0.04 0 0.09 0.06
SNV:-8A 0.07 0.03 0.07 0.02 0.08 0.06
SNV:-3T 0.07 0.03 0.1 0.02 0.03 0.06
SNV:-5T 0.06 0.01 0.09 0.02 0.08 0.06
SNV:-1A 0.06 0.06 0.03 0.07 0.08 0.03
SNV:4A 0.05 0.06 0.05 0.12 0.1 0
SNV:3A 0.04 0.1 0.06 0.11 0.03 0.01
SNV:1T 0.04 0.04 0.06 0.02 0.01 0.04
SNV:-6T 0.03 0.04 0.03 0.04 0.1 0.01
SNV:-7A 0.03 0.01 0.05 0.05 0.02 0.03
SNV:-4T 0.02 0.01 0.03 0.02 0.05 0.01
SNV:-4C 0.03 0.01 0.03 0 0 0.07
SNV:-2C 0.02 0 0 0 0.01 0.01
SNV:2T 0.01 0.04 0.02 0 0.01 0
SNV:-8T 0.01 0.04 0.01 0.04 0.01 0.01
SNV:-3G 0.02 0 0.01 0 0 0
SNV:5A 0.02 0.01 0.01 0 0.01 0.01
SNV:4T 0.01 0.06 0 0 0.02 0
SNV:-3A 0.02 0.03 0 0.02 0 0
SNV:-1T 0.01 0 0 0 0.05 0
SNV:-6C 0.01 0.03 0.01 0 0 0.01
SNV:6A 0.02 0 0.01 0 0.01 0
SNV:-7G 0.01 0 0.02 0.02 0.03 0
SNV:-2T 0.01 0 0.01 0.04 0.01 0
SNV:-5A 0.01 0.01 0.01 0 0 0
SNV:5G 0.01 0 0 0 0.01 0
SNV:-3G,1T 0.01 0 0 0 0.02 0
SNV:-8C 0.01 0 0 0 0.02 0
SNV:1C 0 0.03 0.01 0 0 0
SNV:3T 0 0 0 0.02 0 0.03
SNV:-5G 0 0 0 0.02 0 0
SNV:1T,5A 0 0.03 0 0 0 0
SNV:-5A,1T 0 0 0.01 0.02 0 0
SNV:-4G,1G 0 0 0 0 0 0.03
-19:1D 0.04 0 0.04 0.07 0.04 0.06
2:1D 0.02 0.01 0.05 0 0.05 0.01
-27:1D 0.02 0.01 0.06 0 0.01 0
-30:1D 0.02 0 0 0 0.01 0.03
5:1D 0.01 0.03 0 0 0.04 0
-13:1D 0 0.01 0 0 0.03 0
14:1D 0 0 0.01 0 0 0.03
18:1D 0 0 0.02 0 0 0
-30 -25 -20 -15 -10 -5 -1 1 5 10 15 20
Parentals
Uninjected
dCas9
Cas9enzyme
Cas9RNA
Scrambled
Percentage
0
25
50
75
100

suclg1:

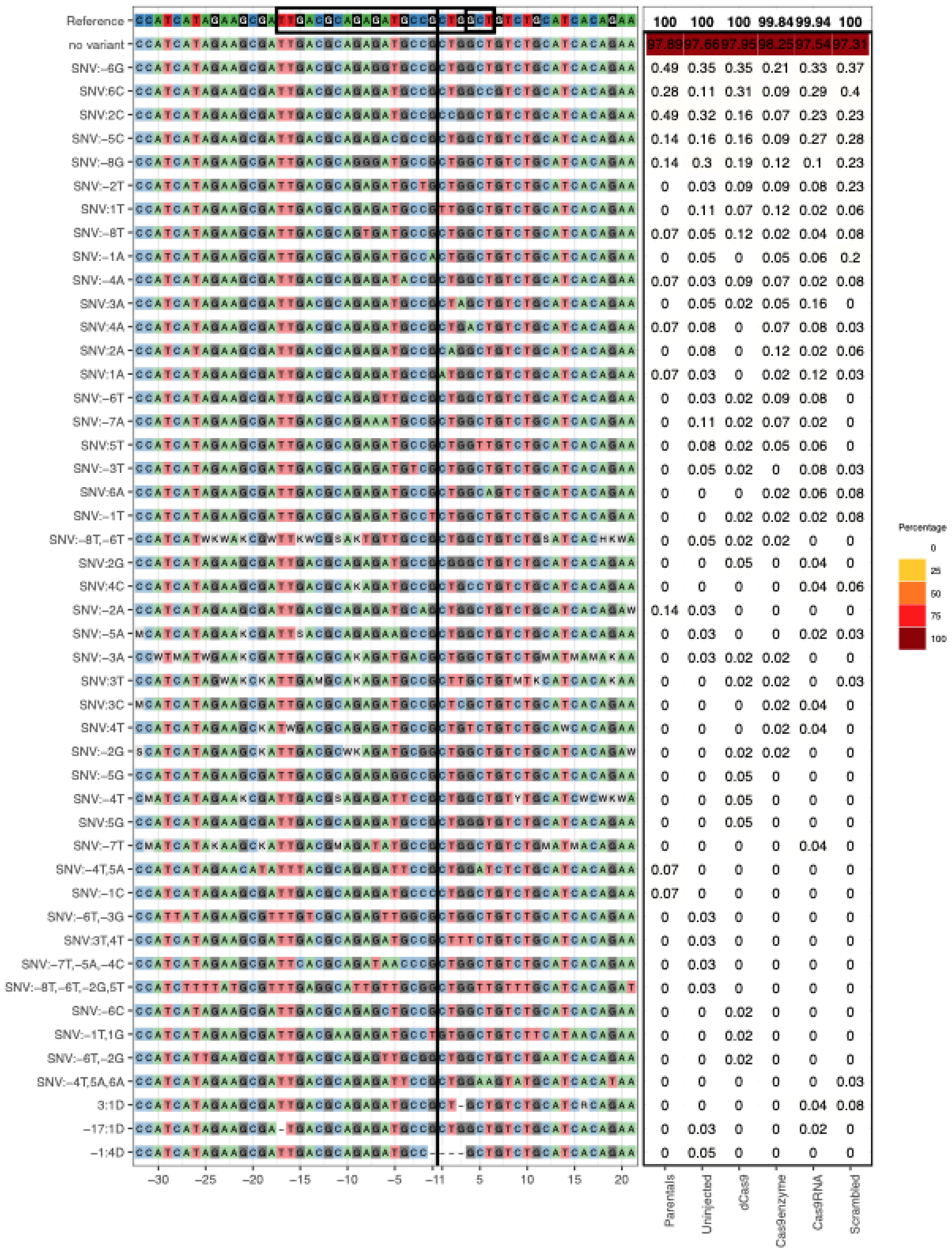
Reference 100 100 100 99.84 99.94 100
no variant 97.89 97.66 97.95 98.25 97.54 97.31
SNV:-6G 0.49 0.35 0.35 0.21 0.33 0.37
SNV:6C 0.28 0.11 0.31 0.09 0.29 0.4
SNV:2C 0.49 0.32 0.16 0.07 0.23 0.23
SNV:-5C 0.14 0.16 0.16 0.09 0.27 0.28
SNV:-8G 0.14 0.3 0.19 0.12 0.1 0.23
SNV:-2T 0 0.03 0.09 0.09 0.08 0.23
SNV:1T 0 0.11 0.07 0.12 0.02 0.06
SNV:-8T 0.07 0.05 0.12 0.02 0.04 0.08
SNV:-1A 0 0.05 0 0.05 0.06 0.2
SNV:-4A 0.07 0.03 0.09 0.07 0.02 0.08
SNV:3A 0 0.05 0.02 0.05 0.16 0
SNV:4A 0.07 0.08 0 0.07 0.08 0.03
SNV:2A 0 0.08 0 0.12 0.02 0.06
SNV:1A 0.07 0.03 0 0.02 0.12 0.03
SNV:-6T 0 0.03 0.02 0.09 0.08 0
SNV:-7A 0 0.11 0.02 0.07 0.02 0
SNV:5T 0 0.08 0.02 0.05 0.06 0
SNV:-3T 0 0.05 0.02 0 0.08 0.03
SNV:6A 0 0 0 0.02 0.06 0.08
SNV:-1T 0 0 0.02 0.02 0.02 0.08
SNV:-8T,-6T 0 0.05 0.02 0.02 0 0
SNV:2G 0 0 0.05 0 0.04 0
SNV:4C 0 0 0 0 0.04 0.06
SNV:-2A 0.14 0.03 0 0 0 0
SNV:-5A 0 0.03 0 0 0.02 0.03
SNV:-3A 0 0.03 0.02 0.02 0 0
SNV:3T 0 0 0.02 0.02 0 0.03
SNV:3C 0 0 0 0.02 0.04 0
SNV:4T 0 0 0 0.02 0.04 0
SNV:-2G 0 0 0.02 0.02 0 0
SNV:-5G 0 0 0.05 0 0 0
SNV:-4T 0 0 0.05 0 0 0
SNV:5G 0 0 0.05 0 0 0
SNV:-7T 0 0 0 0 0.04 0
SNV:-4T,5A 0.07 0 0 0 0 0
SNV:-1C 0.07 0 0 0 0 0
SNV:-6T,-3G 0 0.03 0 0 0 0
SNV:3T,4T 0 0.03 0 0 0 0
SNV:-7T,-5A,-4C 0 0.03 0 0 0 0
SNV:-8T,-6T,-2G,5T 0 0.03 0 0 0 0
SNV:-6C 0 0 0.02 0 0 0
SNV:-1T,1G 0 0 0.02 0 0 0
SNV:-6T,-2G 0 0 0.02 0 0 0
SNV:-4T,5A,6A 0 0 0 0 0 0.03
3:1D 0 0 0 0 0.04 0.08
-17:1D 0 0.03 0 0 0.02 0
-1:4D 0 0.05 0 0 0 0
-30 -25 -20 -15 -10 -5 -1 1 5 10 15 20
Parentals
Uninjected
dCas9
Cas9enzyme
Cas9RNA
Scrambled
Percentage
0
25
50
75
100

tmem176:

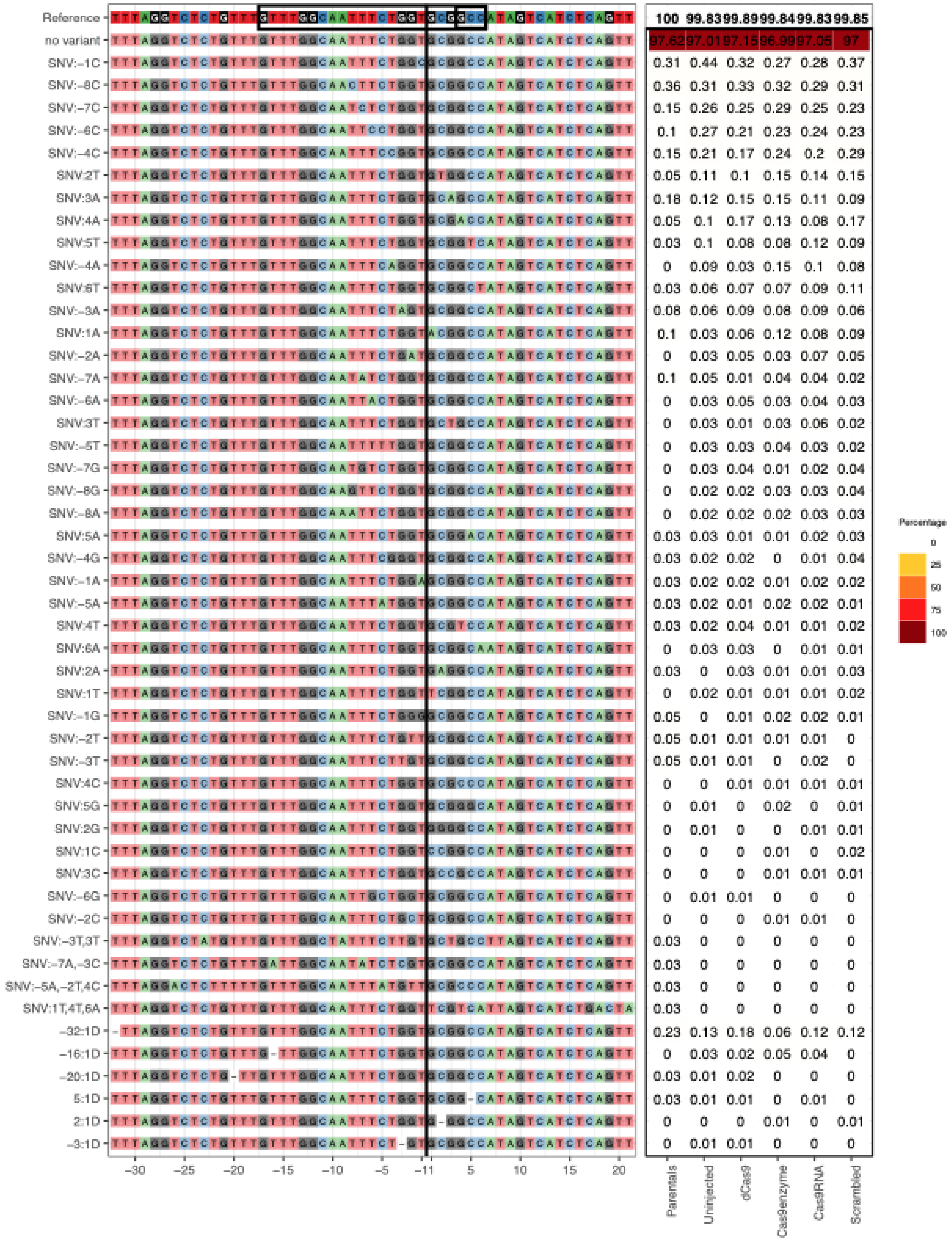
Reference 100 99.83 99.89 99.84 99.83 99.85
no variant 97.62 97.01 97.15 96.99 97.05 97
SNV:-1C 0.31 0.44 0.32 0.27 0.28 0.37
SNV:-8C 0.36 0.31 0.33 0.32 0.29 0.31
SNV:-7C 0.15 0.26 0.25 0.29 0.25 0.23
SNV:-6C 0.1 0.27 0.21 0.23 0.24 0.23
SNV:-4C 0.15 0.21 0.17 0.24 0.2 0.29
SNV:2T 0.05 0.11 0.1 0.15 0.14 0.15
SNV:3A 0.18 0.12 0.15 0.15 0.11 0.09
SNV:4A 0.05 0.1 0.17 0.13 0.08 0.17
SNV:5T 0.03 0.1 0.08 0.08 0.12 0.09
SNV:-4A 0 0.09 0.03 0.15 0.1 0.08
SNV:6T 0.03 0.06 0.07 0.07 0.09 0.11
SNV:-3A 0.08 0.06 0.09 0.08 0.09 0.06
SNV:1A 0.1 0.03 0.06 0.12 0.08 0.09
SNV:-2A 0 0.03 0.05 0.03 0.07 0.05
SNV:-7A 0.1 0.05 0.01 0.04 0.04 0.02
SNV:-6A 0 0.03 0.05 0.03 0.04 0.03
SNV:3T 0 0.03 0.01 0.03 0.06 0.02
SNV:-5T 0 0.03 0.03 0.04 0.03 0.02
SNV:-7G 0 0.03 0.04 0.01 0.02 0.04
SNV:-8G 0 0.02 0.02 0.03 0.03 0.04
SNV:-8A 0 0.02 0.02 0.02 0.03 0.03
SNV:5A 0.03 0.03 0.01 0.01 0.02 0.03
SNV:-4G 0.03 0.02 0.02 0 0.01 0.04
SNV:-1A 0.03 0.02 0.02 0.01 0.02 0.02
SNV:-5A 0.03 0.02 0.01 0.02 0.02 0.01
SNV:4T 0.03 0.02 0.04 0.01 0.01 0.02
SNV:6A 0 0.03 0.03 0 0.01 0.01
SNV:2A 0.03 0 0.03 0.01 0.01 0.03
SNV:1T 0 0.02 0.01 0.01 0.01 0.02
SNV:-1G 0.05 0 0.01 0.02 0.02 0.01
SNV:-2T 0.05 0.01 0.01 0.01 0.01 0
SNV:-3T 0.05 0.01 0.01 0 0.02 0
SNV:4C 0 0 0.01 0.01 0.01 0.01
SNV:5G 0 0.01 0 0.02 0 0.01
SNV:2G 0 0.01 0 0 0.01 0.01
SNV:1C 0 0 0 0.01 0 0.02
SNV:3C 0 0 0 0.01 0.01 0.01
SNV:-6G 0 0.01 0.01 0 0 0
SNV:-2C 0 0 0 0.01 0.01 0
SNV:-3T,3T 0.03 0 0 0 0 0
SNV:-7A,-3C 0.03 0 0 0 0 0
SNV:-5A,-2T,4C 0.03 0 0 0 0 0
SNV:1T,4T,6A 0.03 0 0 0 0 0
-32:1D 0.23 0.13 0.18 0.06 0.12 0.12
-16:1D 0 0.03 0.02 0.05 0.04 0
-20:1D 0.03 0.01 0.02 0 0 0
5:1D 0.03 0.01 0.01 0 0.01 0
2:1D 0 0 0 0.01 0 0.01
-3:1D 0 0.01 0.01 0 0 0
-30 -25 -20 -15 -10 -5 -1 1 5 10 15 20
Parentals
Uninjected
dCas9
Cas9enzyme
Cas9RNA
Scrambled
Percentage
0
25
50
75
100

vps37c:

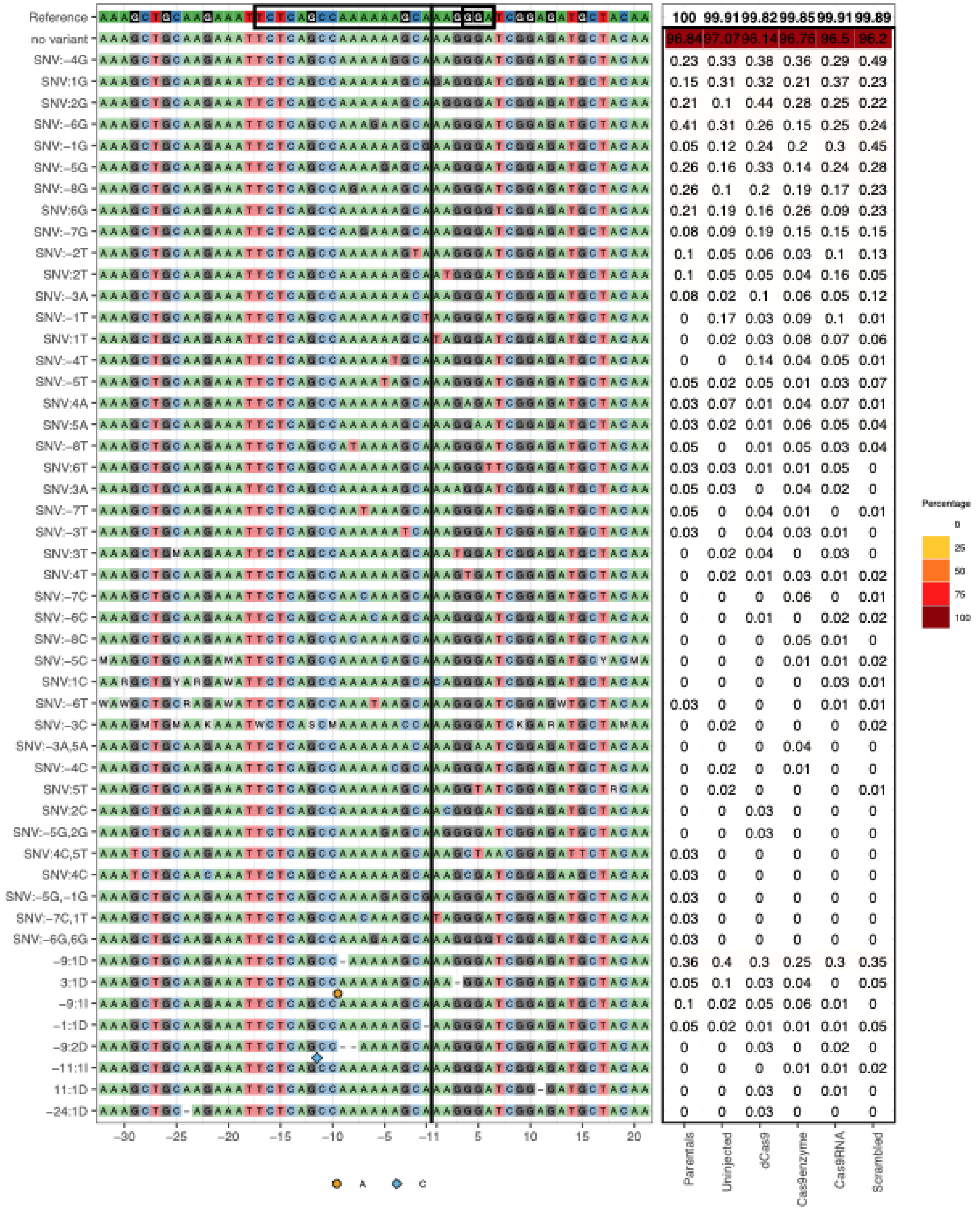
Reference
100 99.91 99.82 99.85 99.91 99.89
no variant 96.84 97.07 96.14 96.76 96.5 96.2
SNV:−4G 0.23 0.33 0.38 0.36 0.29 0.49
SNV:1G 0.15 0.31 0.32 0.21 0.37 0.23
SNV:2G 0.21 0.1 0.44 0.28 0.25 0.22
SNV:−6G 0.41 0.31 0.26 0.15 0.25 0.24
SNV:−1G 0.05 0.12 0.24 0.2 0.3 0.45
SNV:−5G 0.26 0.16 0.33 0.14 0.24 0.28
SNV:−8G 0.26 0.1 0.2 0.19 0.17 0.23
SNV:6G 0.21 0.19 0.16 0.26 0.09 0.23
SNV:−7G 0.08 0.09 0.19 0.15 0.15 0.15
SNV:−2T 0.1 0.05 0.06 0.03 0.1 0.13
SNV:2T 0.1 0.05 0.05 0.04 0.16 0.05
SNV:−3A 0.08 0.02 0.1 0.06 0.05 0.12
SNV:−1T 0 0.17 0.03 0.09 0.1 0.01
SNV:1T 0 0.02 0.03 0.08 0.07 0.06
SNV:−4T 0 0 0.14 0.04 0.05 0.01
SNV:−5T 0.05 0.02 0.05 0.01 0.03 0.07
SNV:4A 0.03 0.07 0.01 0.04 0.07 0.01
SNV:5A 0.03 0.02 0.01 0.06 0.05 0.04
SNV:−8T 0.05 0 0.01 0.05 0.03 0.04
SNV:6T 0.03 0.03 0.01 0.01 0.05 0
SNV:3A 0.05 0.03 0 0.04 0.02 0
SNV:−7T 0.05 0 0.04 0.01 0 0.01
SNV:−3T 0.03 0 0.04 0.03 0.01 0
SNV:3T 0 0.02 0.04 0 0.03 0
SNV:4T 0 0.02 0.01 0.03 0.01 0.02
SNV:−7C 0 0 0 0.06 0 0.01
SNV:−6C 0 0 0.01 0 0.02 0.02
SNV:−8C 0 0 0 0.05 0.01 0
SNV:−5C 0 0 0 0.01 0.01 0.02
SNV:1C 0 0 0 0 0.03 0.01
SNV:−6T 0.03 0 0 0 0.01 0.01
SNV:−3C 0 0.02 0 0 0 0.02
SNV:−3A,5A 0 0 0 0.04 0 0
SNV:−4C 0 0.02 0 0.01 0 0
SNV:5T 0 0.02 0 0 0 0.01
SNV:2C 0 0 0.03 0 0 0
SNV:−5G,2G 0 0 0.03 0 0 0
SNV:4C,5T 0.03 0 0 0 0 0
SNV:4C 0.03 0 0 0 0 0
SNV:−5G,−1G 0.03 0 0 0 0 0
SNV:−7C,1T 0.03 0 0 0 0 0
SNV:−6G,6G 0.03 0 0 0 0 0
−9:1D 0.36 0.4 0.3 0.25 0.3 0.35
3:1D 0.05 0.1 0.03 0.04 0 0.05
−9:1I 0.1 0.02 0.05 0.06 0.01 0
−1:1D 0.05 0.02 0.01 0.01 0.01 0.05
−9:2D 0 0 0.03 0 0.02 0
−11:1I 0 0 0 0.01 0.01 0.02
11:1D 0 0 0.03 0 0.01 0
−24:1D 0 0 0.03 0 0 0
−30 −25 −20 −15 −10 −5 −1 1 5 10 15 20
Parentals
Uninjected
dCas9
Cas9enzyme
Cas9RNA
Scrambled
A
C
Percentage
0
25
50
75
100

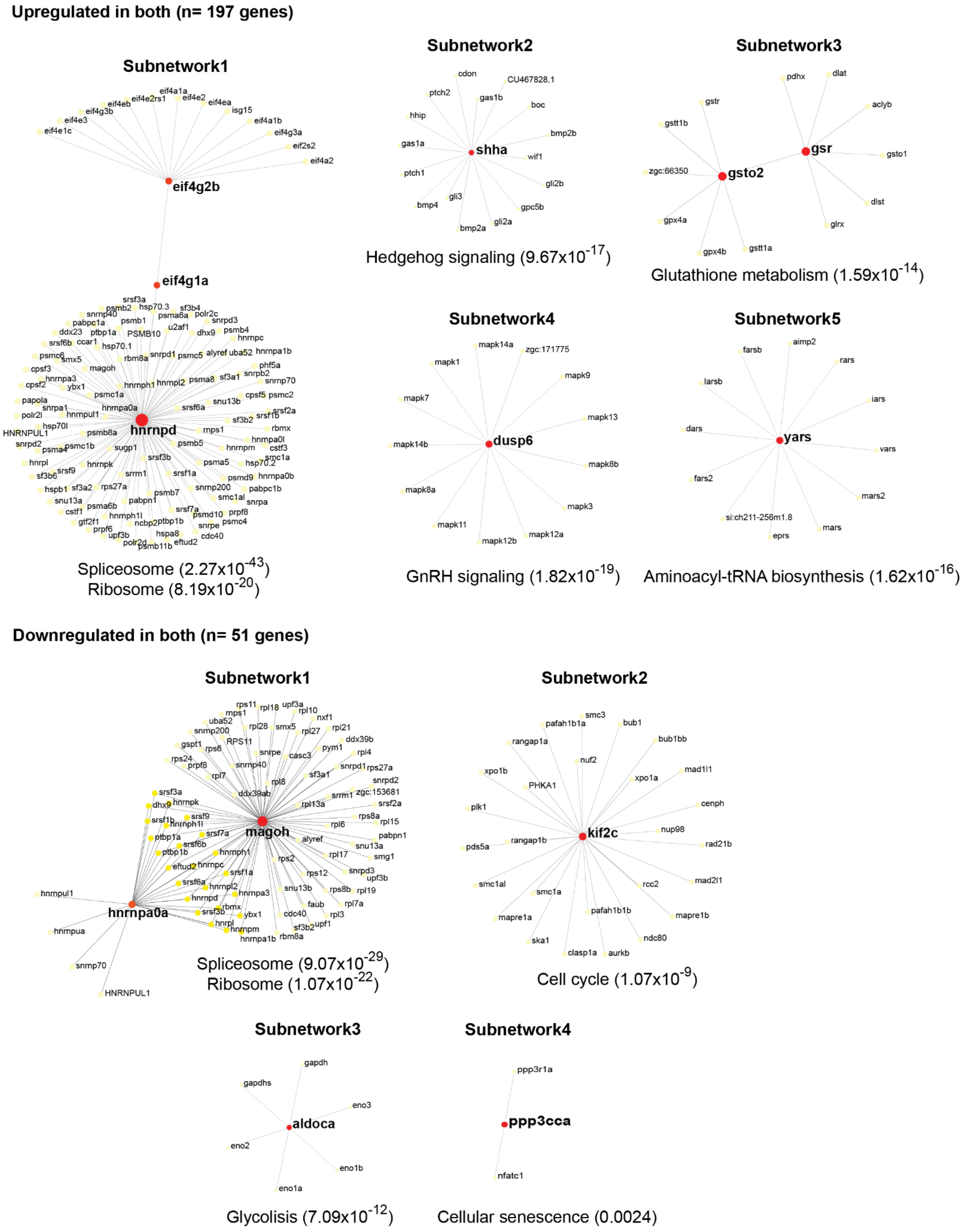

Supplementary Figure 3. **Network analyses of the shared differentially expressed genes in Cas9 enzyme and Cas9 mRNA injected larvae.** Each subnetwork includes the top enriched KEGG pathways below with the adjusted *p-values* in parentheses [75]. Network analyses were performed using the Network Analyst online tool (www.networkanalyst.ca).

Supplementary Tables Legends

Supplementary Table 1. Oligonucleotides utilized for all assays performed throughout the project.

Supplementary Table 2. On-target efficiency scores for the 50 gRNAs obtained from different *in silico* prediction tools, empirical cleavage scores *in vivo* using Sanger sequencing and sequence deconvolution tools (TIDE and ICE), from the *in vitro* assay CIRCLE-seq (RPMN), and Illumina sequencing using the CrispRVariants software. RPMN: reads per million normalized (see Methods).

Supplementary Table 3. Description of the off-targets predicted by CRISPRScan and CIRCLE-seq for the 50 evaluated gRNAs. The percentage of sites intersecting genes from the CRISPRScan tool was obtained from the top 30 predicted off-targets since the tool only provides detailed information from these.

Supplementary Table 4. List of the frameshift variants identified in protein-coding genes in samples injected with either Cas9 enzyme or Cas9 mRNA using uninjected batch-siblings as reference (see Methods).

Supplementary Table 5. Illumina mosaicism percentages observed in larvae injected with Cas9 (enzyme or mRNA), catalytically dead Cas9 (dCas9), scrambled gRNA, uninjected batch siblings, and a finclip from their crossing parents.

Supplementary Table 6. List of differentially expressed genes observed in Cas9-injected samples relative to uninjected batch-siblings and siblings from another batch.

Supplementary Table 7. List of gene ontology terms enriched in upregulated genes found in both injection treatments (Cas9 enzyme and Cas9 mRNA).
